# *Pten* knockout affects drug resistance differently in melanoma and kidney cancer

**DOI:** 10.1007/s43440-023-00523-y

**Published:** 2023-09-06

**Authors:** Klaudia Brodaczewska, Aleksandra Majewska, Aleksandra Filipiak-Duliban, Claudine Kieda

**Affiliations:** 1 Laboratory of Molecular Oncology and Innovative Therapies, Military Institute of Medicine – National Research Institute, Szaserów 128, 01-141 Warsaw, Poland; 2 Postgraduate School of Molecular Medicine (Medical University of Warsaw), Żwirki I Wigury 61, 02-091 Warsaw, Poland; 3 Center for Molecular Biophysics UPR 4301, CNRS, 45071 Orleans, France

**Keywords:** Cisplatin, Melanoma, PAI-1, PTEN, RCC

## Abstract

**Background:**

PTEN is a tumor suppressor that is often mutated and nonfunctional in many types of cancer. The high heterogeneity of PTEN function between tumor types makes new *Pten* knockout models necessary to assess its impact on cancer progression and/or treatment outcomes.

**Methods:**

We aimed to show the effect of CRISPR/Cas9-mediated *Pten* knockout on murine melanoma (B16 F10) and kidney cancer (Renca) cells. We evaluated the effect of PTEN deregulation on tumor progression in vivo and in vitro, as well as on the effectiveness of drug treatment in vitro. In addition, we studied the molecular changes induced by *Pten* knockout.

**Results:**

In both models, *Pten* mutation did not cause significant changes in cell proliferation in vitro or in vivo. Cells with *Pten* knockout differed in sensitivity to cisplatin treatment: in B16 F10 cells, the lack of PTEN induced sensitivity and, in Renca cells, resistance to drug treatment. Accumulation of pAKT was observed in both cell lines, but only Renca cells showed upregulation of the p53 level after *Pten* knockout. PTEN deregulation also varied in the way that it altered PAI-1 secretion in the tested models, showing a decrease in PAI-1 in B16 F10 *Pten/KO* and an increase in Renca *Pten/KO* cells. In kidney cancer cells, *Pten* knockout caused changes in epithelial to mesenchymal transition marker expression, with downregulation of E-cadherin and upregulation of Snail, *Mmp9*, and *Acta2* (α-SMA).

**Conclusions:**

The results confirmed heterogenous cell responses to PTEN loss, which may lead to a better understanding of the role of PTEN in particular types of tumors and points to PTEN as a therapeutic target for personalized medicine.

**Supplementary Information:**

The online version contains supplementary material available at 10.1007/s43440-023-00523-y.

## Introduction

PTEN (phosphatase and tensin homolog deleted from chromosome 10) is an important factor that regulates many of the processes related to tumor development and progression. It is estimated that approximately 13.5% of human cancers have PTEN-altered function or mutation [1]. The dysregulation of PTEN activity can be associated with many factors, including genetic alteration, post-transcriptional and post-translational modifications, or interactions with other proteins [2]. The main role of PTEN is associated with its lipid phosphatase activity, which acts as a negative regulator of PI3K/AKT signaling, affecting many basic processes of survival, growth, proliferation, angiogenesis, metabolism, and migration [3–5]. PTEN can also act in a lipid-phosphatase-independent manner, which is related to its localization in the cell [6]; nuclear PTEN affects DNA repair, cell cycle regulation, and chromosome stability [7, 8]. PTEN is known to interact with the other main tumor suppressor, p53. PTEN–p53 mutual regulation may occur at the transcriptional and protein levels, affecting major processes in cancer progression [8, 9]. Multifunctional PTEN activity is also crucial in modulating the tumor microenvironment (TME), affecting not only cancer cells but also additional features of the TME—immune response and angiogenesis [10, 11].

The diversity of PTEN cellular locations, corresponding to distinct functions with consequences for tumor progression, together with the possibility of various modifications of its expression and activity, makes the prognostic value of PTEN largely unknown [12]. Substantial evidence indicates that low PTEN levels correlate with poorer patient survival rates. In melanoma patients, the loss of PTEN expression correlates significantly with decreased overall survival and a shorter time to brain metastasis formation [13]. Similar results have been documented in other types of cancer, where PTEN loss was associated with increased aggressiveness, metastasis, and poorer prognosis (breast cancer [14], ovarian cancer [15] and hepatocellular carcinoma [16]). In glioblastoma, PTEN levels affected tumor differentiation and prognosis, but the impact of *PTEN* mutations was restricted to highly malignant tumors only [17]. Inconsistencies have also been found in kidney cancer—two independent meta-analyses showed different results: a significant effect of PTEN levels on tumor progression [18] and a low predictive value [19] in renal cell carcinoma (RCC) patients.

In addition to the effects of PTEN on cancer progression, it is also known to modulate sensitivity to different types of treatment. *PTEN* mutations caused resistance to radiotherapy and chemotherapy of prostate cancer cells by hyperactivating the AKT pathway [20]. In non-small cell lung carcinoma (NSCLC) models, PTEN loss contributed to radio-resistance, affecting the signaling pathways of DNA damage [21]. Resistance to cisplatin, a chemotherapeutic that causes DNA damage-mediated apoptotic signals, was observed in ovarian cancer cells after *PTEN* knockout (KO) [22]. A *PTEN* mutation in endometrial cancer cells resulted in drug resistance to docetaxel, a cell division inhibitor [23]. The loss of PTEN caused resistance to apoptosis by activating the anti-apoptotic mechanisms mediated by MDM2 in acute lymphoblastic leukemia models [24]. In kidney cancer, PTEN alteration affected resistance to the tyrosine kinase inhibitors (TKIs) sunitinib and sorafenib, drugs primarily targeting tumor angiogenesis [25]. In melanoma, it was reported that PTEN loss promoted immune resistance and caused inferior outcomes of PD-1 (programmed death-1) inhibitor therapy [26]. PTEN’s miscellaneous roles in the response to various treatments are strictly related to its multifunctionality in targeting distinct signaling pathways and cellular processes.

Thus, the high heterogeneity of tumor responses to PTEN dysregulation and its importance in key tumor progression processes make new *PTEN* knockout models necessary. Here, we aimed to establish stable murine melanoma B16 F10 and kidney cancer Renca cells with a loss of PTEN function to investigate the significance of this manipulation in tumor progression, molecular changes, and responses to treatment.

## Materials and methods

### Cell lines

Murine kidney cancer cells (Renca) were purchased from ATCC (Cat# CRL-2947, LOT# 63,226,315 ATCC, USA). Murine melanoma cells (B16 F10) were kindly gifted by Prof. Józef Dulak from the Department of Medical Biotechnology, Faculty of Biochemistry, Biophysics, and Biotechnology, Jagiellonian University, Cracow, Poland (authenticated by the ATCC Cell Authentication Service in 2021). The profiles of the B16 F10 samples were the same in 97% of cases as the reference profile ATCC MUSA0830. Both cell lines were cultured in RPMI-1640 GlutaMax™ medium (Thermo Fisher Scientific, Waltham, MA, USA), with 10% fetal bovine serum (FBS) (Thermo Fisher Scientific, Waltham, MA, USA) and regularly checked for the presence of mycoplasma using PCR assay (Biomedica, Poland).

The CRISPR/Cas9 system was used to knock out *Pten* expression in melanoma cells using the same protocol as that used in the Renca cell line that was described previously [27]. The same pSpCas9(BB)-2A-Puro(PX459)V2.0 (Gene Script, Piscataway, NJ, USA) plasmids, containing gRNAs targeting *Pten* (gRNA1: CCAATTCAGGACCCACGCGGCGG, gRNA2:GAACTGTCCTCCCGCCG-CGTGG), were used to transfect the B16 F10 cells. Cas9 nuclease only was used as a control (WT-wild type); the cells were transfected with empty plasmid and treated with the same protocol as the *Pten*-modified cells.

The cells were seeded in 24-well plates 24 h prior to transfection (1.25 × 10^4^ per well), allowing them to adhere to the surface of the well. Five hours before transfection, the cells were starved with a medium without FBS. Transfections were performed using Lipofectamine 2000 transfection reagent (Thermo Fisher Scientific, Waltham, MA, USA) according to the manufacturer’s protocol. The selection of plasmid-containing cells was performed using puromycin (5 µg/mL; Thermo Fisher Scientific, Waltham, MA, USA; concentration established in preliminary experiments as effective for elimination of both cell lines) starting 5 h after transfection and continued for another 48 h. Cells treated with Lipofectamine 2000 only served as a control for selection with puromycin. Surviving cells transfected with *Pten* or control plasmids were used to limit dilution cloning. A single clone where *Pten* knockout was confirmed by no detection of protein using western blotting and the sequencing of the exon 7 fragment was selected. A WT control clone was selected randomly and sequenced to confirm no effect on the *Pten* gene. Detailed sequencing data of the obtained clones were prepared using the Mutation Surveyor® software and are presented in Supplementary Fig. 1. The sequences of both types of cells were compared to the original cells before transfection (termed Renca and B16 F10). Cells with *Pten* knockout are henceforth referred to as *Pten/KO* cells, while negative controls (transfected with empty plasmids) are referred to as *Pten/WT*.

### In vivo* experiments*

To verify the effect of *Pten* knockout on tumor growth in vivo, Renca or B16 F10 cells in the *Pten/WT* and *Pten/KO* variants were implanted subcutaneously into the legs of mice—Renca into BALB/c and B16 F10 into C57BL6, respectively. The mice were obtained from the Medical University of Bialystok, Poland. The animal care and experimental procedures were approved by the Second Warsaw Local Ethics Committee for Animal Experimentation (approval no. WAW2/76/2017) and performed following Directive 2010/63/EU regulations. The mice were housed in a controlled environment (12 h light / 12 h dark cycle) with ad libitum access to tap water and a fully-fledged diet.

Details about the Renca cells implanted into BALB/c mice have been shown previously [27]. Melanoma cells—B16 F10 (2 × 10^5^ cells) with Matigel™ (Corning, NY, USA) diluted 1:3 in PBS—were implanted into the legs of six- to eight-week-old female C57BL6 mice as subcutaneous tumors. After 22 days of tumor growth, the mice were euthanized, and the tumors were weighed and measured. Fragments of tumor tissue were used for RNA isolation. The experiment was performed using two separate sets of animals, each containing four mice.

### Assessment of susceptibility to treatment

The *Pten/WT* and *Pten/KO* cells of both tested models were cultured in standard conditions: 37 °C; 21% pO_2_; 5% CO_2_. To assess the resistance to cisplatin (Sigma-Aldrich, Darmstadt, Germany) and sunitinib (Sigma-Aldrich, Darmstadt, Germany) treatments, experiments were performed in 96-well plates. Cells were seeded (5 000 cells per well Renca; 1 500 cells per well B16 F10) and cultured for 24 h, and the medium was exchanged to remove unadhered cells. After an additional 24 h, drugs were added at their final concentrations—cisplatin: 2.50, 3.75, 5.0, 7.5, 10.0, 15.0, 20.0, and 24.0 µM; sunitinib: 1.875, 2.5, 3.75, 5.0, 7.5, 10.0, and 15.0 µM. Cell viability was checked after 48 h of culture with the drug using Alamar Blue assay (Thermo Fisher Scientific, Waltham, MA, USA) according to the manufacturer’s protocol. Fluorescence was measured using a VarioScan Lux (Thermo Fisher Scientific, Waltham, MA, USA), and the results are presented as a percentage of the untreated control. The IC50 dose (half-maximal inhibitory concentration) was calculated using GraphPad Prism 9.0.

### Colony formation assay

The soft agar colony formation test was performed on 24-well plates coated with 1.5% agar. Renca cells (*Pten/WT* and *Pten/KO*) were seeded on the 1.5% agar layer at a very low density (900 cells per well resuspended in 0.6% agar in RPMI 1640 10% FBS). A full medium was applied above the cell-agar layer to avoid drying. The cells were cultured for another three weeks under standard oxygen conditions: 37 °C; 21% pO_2_; 5% CO_2_. The formed colonies were fixed and stained with crystal violet. The number and average size (diameter [cm]) of the colonies were estimated using ImageJ software.

### Protein detection using western blot

Proteins for western blot were collected from cells cultured in T75 flasks, detached with Accutase solution (Biolegend, USA), washed twice with PBS, and lysed with RIPA buffer containing Cocktail inhibitors (both from Thermo Fisher Scientific, Waltham, MA, USA). Total protein concentration was assessed by BCA assay. Twelve micrograms (12 µg) of protein were solubilized in a Laemmli sample buffer (AlfaAesar, Haverhill, MA, USA), separated on 12% polyacrylamide gel, and transferred onto nitrocellulose membranes (BioRad, Hercules, CA,USA). Proteins were detected on the membranes using Ponceau S staining. Nonspecific binding was diminished by a blocking step in 5% skimmed milk (2 h; room temperature). Membranes were incubated overnight at 4 °C in the solution of primary antibodies (Table 1) and then incubated for a further 2 h at room temperature, with the relevant secondary antibody conjugated with horseradish peroxidase (HRP) (Table 1). Bands were detected using Luminol as an HRP substrate (Santa-Cruz, CA, USA) with X-ray films. Quantification of the integrated optical density (IOD) of the bands was calculated using ImageJ software and normalized to the IOD of the loading control protein Vinculin.

**Table 1:** List of antibodies used in Western Blot

Antibody	#Cat Number	Dilution
anti-AKT	#sc-5298, Santa Cruz Biotechnology	1:700
anti-PTEN	#9549, Cell Signalling Technology	1:750
anti-pAKT	#MAB887, R&D System, USA	1:1000
anti-Snail	#3879 Cell Signalling Technology	1:1000
anti-p53	#2524, Cell Signalling Technology	1:1000
anti-E-cadherin	#3195, Cell Signalling Technology	1:1000
anti-Vinculin (loading control)	#sc-59803, Santa Cruz Biotechnology	1:1000
anti-Rabbit IgG Antibody (secondary antibody)	#Pl-1000 Vector Laboratories, USA	1:10 000
anti-Mouse IgG Antibody (secondary antibody)	#Pl-2000 Vector Laboratories, USA	1:10 000

### Gene expression assessment by qRT-PCR

RNA was isolated from fragments of tumor tissue or cells cultured in T75 flasks using the column method (RNeasy Mini Kit; Qiagen, Germany). The samples were freed from DNA using the TURBO DNA-free kit (Thermo Fisher Scientific, USA), and reverse transcription was performed using 2 µg of total RNA for the tumor samples and 1.5 µg for the cell culture samples (High-Capacity cDNA Reverse Transcription Kit; Thermo Fisher Scientific, USA). Real-time PCR was performed using TaqMan™ Gene Expression Master Mix with TaqMan probes (all from Thermo Fisher Scientific, USA; listed in Table 2), or using PowerUp SYBR Master Mix (Thermo Fisher Scientific, USA) with the primers listed in Table 2. Reactions were run on a Bio-Rad CFX384 qPCR system (BioRad, Hercules, CA, USA). The relative mRNA levels were calculated using the 2(-Delta C[T]) method, with normalization to the expression of *β-Actin* as a housekeeping gene.

**Table 2:** List of TaqMan probes and primers sequences used in real-time PCR

	TaqMan probes (Assay ID)	
*Vegfa*	Mm00437306	
*Pten*	Mm00477208	
*Akt1*	Mm00437306	
*p53*	Mm01731287	
*β-Actin*	Mm02619580	
	Primers sequences	
	Forward	Reverse
*Serpine1* (PAI-1)	CCTCCACAGCCTTTGTCATCT	TTCGTCCCAAATGAAGGCGT
*Mmp9*	CAGCCGACTTTTGGTCTTC	CGGTACAAGTATGCCTCTGCCA
*Acta2* (α-SMA)	CTTCGTGACTACTGCCGGAGC	AGGTGGTTTCGTGGATGCC
*β-Actin*	CCTAGGCACCAGGGTGTGA	GTTGGCCTTAGGGTTCAGGG
*Vegfr2*	AAACAAAACTGTAAGTACGCTGGTC	GCAGCAGGTTGCACAGTAATTT

### Detection of VEGF-A and PAI-1 secretion

The levels of VEGF-A (vascular endothelial growth factor A) and PAI-1 (plasminogen activator inhibitor 1) were measured in conditioned media from *Pten*/*WT* and *Pten/KO* B16 F10 and Renca cells using commercially available enzyme-linked immunosorbent assays Mouse VEGF DuoSet ELISA and Mouse PAI-1 DuoSet ELISA (both R&D Systems, USA), according to the manufacturer’s protocol. Concentrations were calculated against the standard curve using recombinant proteins provided in the kits. Absorbance (450 nm) was measured using a VarioScan Lux (Thermo Fisher Scientific, Waltham, MA, USA).

### In vitro* experiments in hypoxic conditions*

To assess *Pten/WT* and *Pten/KO* cells’ susceptibility to cisplatin treatment in hypoxia (1% pO_2_), cells were seeded in 96-well plates under standard oxygen conditions and allowed to adhere to the culture surface. After 24 h, the medium was changed to a pre-equilibrated hypoxic medium, and the cells were cultured in an XVivo X3 workstation (Biospherix, USA) in 1% pO_2_ and 5% CO_2_ at 37 °C for a further 24 h. Next, cisplatin was added at the final testing concentrations, as previously used in normoxic conditions, and Alamar Blue was used, as described above. The conditioned media were collected from cells cultured in hypoxia for 72 h without drugs, and PAI-1 secretion was detected using a Mouse PAI-1 DuoSet ELISA (R&D Systems, USA), as described above.

### Statistical analysis

All statistical analyses were performed using GraphPad Prism 9.0 software. The normality of the data distribution was checked using the Shapiro–Wilk’s test. Student’s t-test or the Mann–Whitney U test were used where applicable. The data are expressed as the mean ± standard error of the mean (SEM) for parametric data or as box plots with medians for non-parametric data. Detailed information is provided in the figure captions.

## Results

### Pten knockout does not affect melanoma or kidney cancer progression

**Fig. 1:**
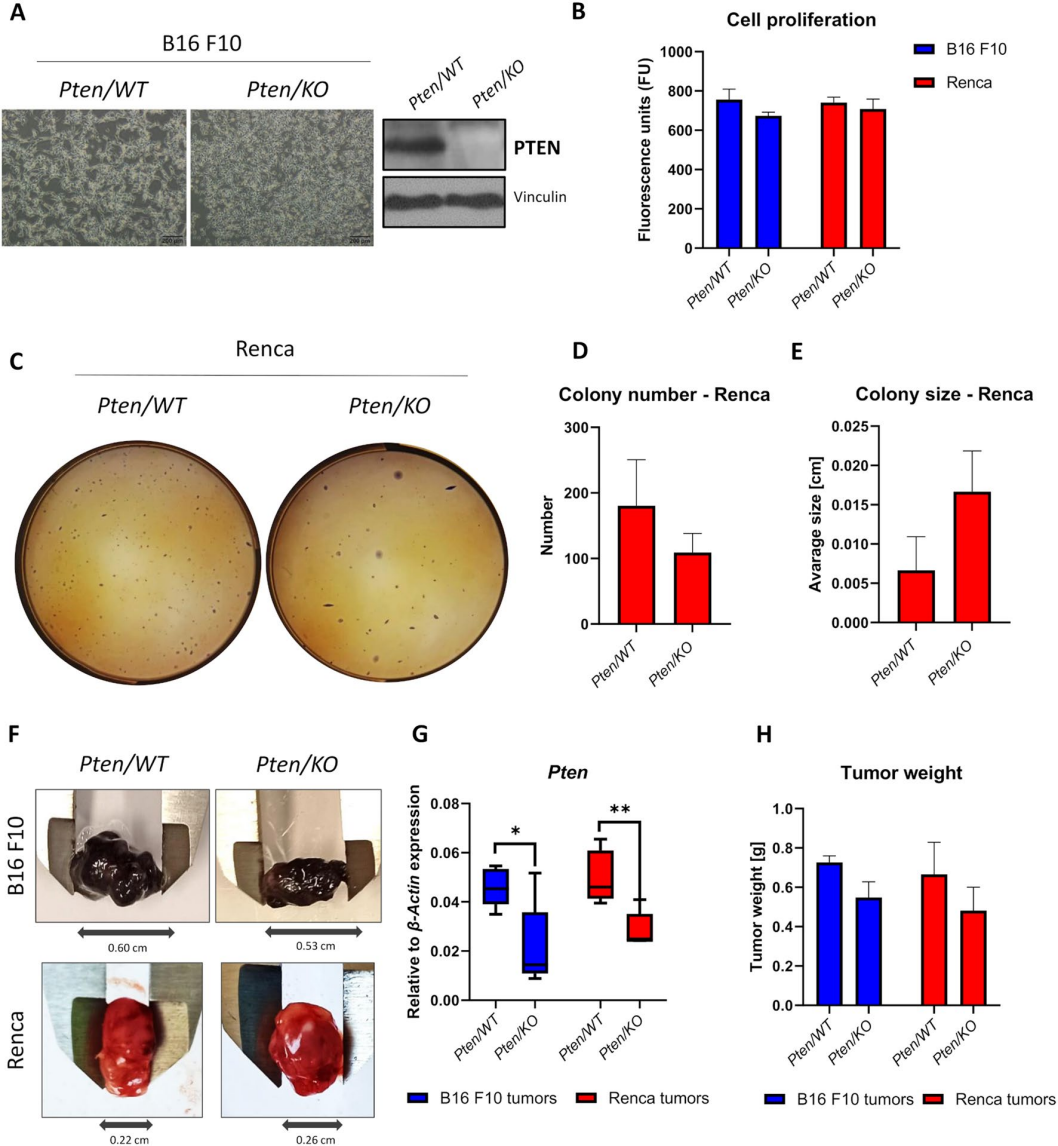
Effect of *Pten* knockout on melanoma and kidney cancer growth. **A** Cell morphology (scale bar: 200 µm) and PTEN protein levels assessed by western blot in B16 F10 *Pten/WT* and *Pten/KO* cells. **B** Cell proliferation measured as mitochondrial activity, after 72 h culture, of B16 F10 and Renca cells with different PTEN statuses, shown as fluorescence units (FU); values are shown as the mean ± SEM; Student’s *t*-test (B16 F10, not significant; Renca, not significant). **C** Representative photos of colony formation by Renca *Pten/WT* and *Pten/KO* cells. **D** Quantification of colony numbers formed by Renca cells with different PTEN statuses; values are shown as the mean ± SEM; Student’s *t*-test (not significant). **E** Quantification of colony size, measured as diameter, formed by Renca cells with different PTEN statuses; values are shown as the mean ± SEM; Student’s *t*-test (not significant). **F** Representative photos of B16 F10 and Renca tumors formed by cells with different PTEN statuses. **G** Box plot represents relative to *β-Actin Pten* expression in *Pten/WT* and *Pten/KO* tumor masses; middle line in box represents the median; Mann–Whitney U test (B16 F10: U = 4, *n*_1_ = *n*_2_ = 6, p-value = 0.0260, two-tailed; Renca: U = 1, *n*_1_ = *n*_2_ = 6, p-value = 0.0043, two-tailed). **H** Weight of tumors formed by B16 F10 and Renca cells with different PTEN statuses; values are shown as the mean ± SEM; Student’s *t*-test (n = 3, not significant)

To determine the effect of PTEN on melanoma and kidney cancer progression, CRISPR/Cas9-mediated *Pten* knockout models were developed. The morphology and PTEN protein levels of the B16 F10 cells are presented in Fig. 1A, while data concerning kidney cancer (Renca) cells were shown previously [27]. In both models, the *Pten* mutation did not cause significant changes in cell proliferation in vitro (Fig. 1B), measured as the changes in fluorescence in the Alamar Blue assay. A lack of *Pten/KO* impact on cell proliferation and survival in kidney cancer cells was also demonstrated in the colony formation assay (Fig. 1C–E); no changes were observed in the size or number of colonies formed in the soft agar.

As the TME is a complex system, and interactions between tumor cells and other components of the TME are important for cancer progression, the effect of *Pten* knockout was also checked in the in vivo models. Subcutaneous B16 F10 or Renca tumors, induced using *Pten/WT* or *Pten/KO* cells, were assessed (Fig. 1F). Despite the presence of other components of the TME, the reduced *Pten* expression was maintained in the tumor mass both at the transcript (Fig. 1G) and protein (Supplementary Figure S2A, B) levels. Although significant changes in PTEN levels were maintained, there was no difference in tumor weight in both tested tumor types (Fig. 1H).

### Pten knockout causes differential changes in cisplatin sensitivity in Renca and B16 F10 cells

**Fig. 2:**
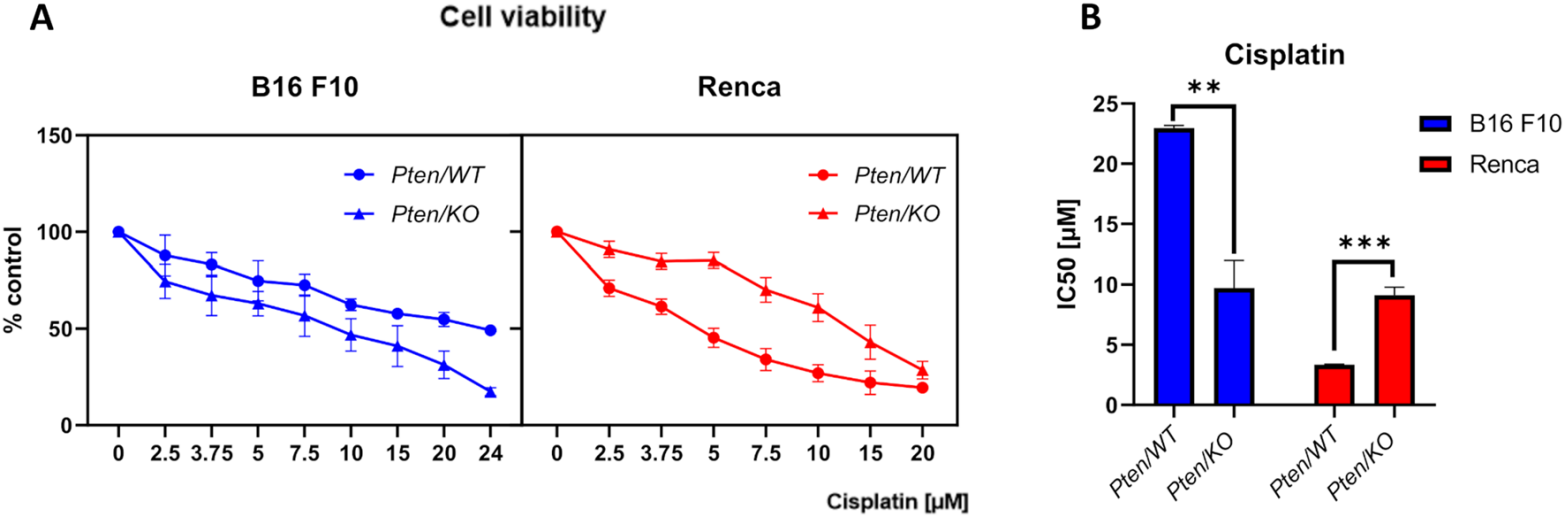
Effect of *Pten* knockout on cisplatin sensitivity in B16 F10 and Renca cells. **A** Viability of B16 F10 and Renca cells with different PTEN statuses after various doses of cisplatin treatment, measured by Alamar Blue, shown as a percentage of untreated control for each PTEN variant normalized to 100%. **B** IC50 dose (half-maximal inhibitory concentration) of cisplatin for different PTEN variant cells; values are shown as the mean ± SEM; Student’s *t*-test (B16 F10: ** p-value = 0.0011, t_6_ = 5.846, Renca: *** p-value = 0.0001, t_6_ = 8.579)

Since no significant changes in the progression of *Pten/KO* tumors were observed, the sensitivity of cells to anticancer treatment was assessed in vitro. We performed initial experiments for drug sensitivity using cisplatin and sunitinib, which represent different models of action. Only cisplatin sensitivity was affected by *Pten* knockout. In the melanoma model, *P ten/KO* cells showed lower resistance to cisplatin treatment than *Pten/WT* cells in the whole range of tested concentrations (Fig. 2A). Based on this, the calculated IC50 dose was almost two times lower for B16 F10 *Pten/KO* (Fig. 2B). An inverse relationship was observed in the kidney cancer model—the IC50 dose was higher for *Pten*-mutated cells compared to wild-type cells (Fig. 2B). No significant changes in viability were observed for sunitinib (Supplementary Figure S3A, B), which suggests that the effect of *Pten* knockout on cell sensitivity to drugs is closely related to the mechanism of drug action. However, the differences between the two tested cancer types may be related to the distinct modulation of signaling pathways after *Pten* knockout.

### Pten knockout induces distinct molecular changes in renal cell carcinoma and melanoma

**Fig. 3:**
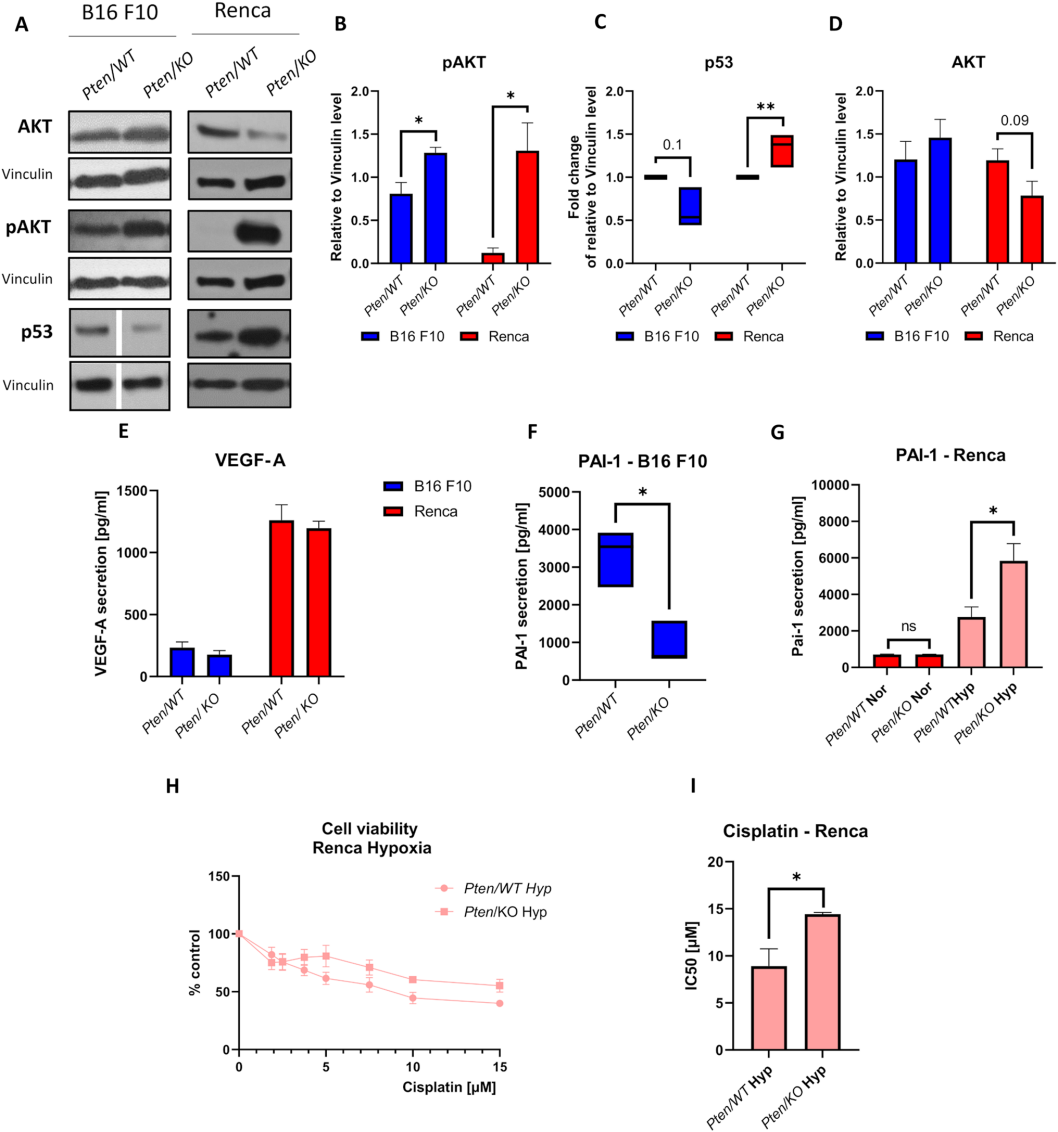
Effect of *Pten* knockout on molecular changes and secretory factors in melanoma and kidney cancer models. **A** AKT, pAKT, and p53 detection by western blot, with Vinculin as loading control, in B16 F10 and Renca with different PTEN statuses. The gap between *Pten/WT* and *Pten/KO* shows that samples on the gel were in a different order and were rearranged for the figure. **B** pAKT level relative to Vinculin in B16 F10 and Renca cells with different PTEN statuses; values are shown as the mean ± SEM; Student’s *t*-test (B16 F10: * p-value = 0.0167, t_6_ = 8.579; Renca: * p-value = 0.021, t_4_ = 3.285). **C** Box plot represents fold change of p53 level relative to Vinculin in *Pten/KO* cells compared to *Pten/WT* cells normalized to 1; middle line in box represents the median; Mann–Whitney U test (B16 F10: U = 0, *n*_1_ = *n*_2_ = 3, p-value = 0.100, two-tailed; Renca: U = 0, *n*_1_ = *n*_2_ = 5, * p-value = 0.0079, two-tailed). **D** AKT level relative to Vinculin in B16 F10 and Renca cells with different PTEN statuses; values are shown as the mean ± SEM; Student’s *t*-test (B16 F10: not significant; Renca: p-value = 0.0911, t_8_ = 1.920). **E** VEGF-A secretion by B16 F10 and Renca cells with different PTEN statuses, measured by ELISA; values are shown as the mean ± SEM; Student’s *t*-test (B16 F10, not significant; Renca, not significant). **F** Box plot of PAI-1 secretion by B16 F10 cells with different PTEN statuses, measured by ELISA; middle line in box represents the median; Mann–Whitney U test (U = 0, *n*_1_ = *n*_2_ = 4, * p-value = 0. 0286, two-tailed). **G** PAI-1 secretion by Renca cells with different PTEN statuses cultured in normoxia and hypoxia, measured by ELISA; values are shown as the mean ± SEM; Student’s *t*-test (normoxia: n = 4, not significant; hypoxia: * p-value = 0.03, t_6_ = 2.830). **H** Viability of Renca *Pten/WT* and *Pten/KO* cells after various doses of cisplatin in hypoxic conditions, measured by Alamar Blue, shown as a percentage of the untreated control for each PTEN variant. **I** IC50 dose (half-maximal inhibitory concentration) of cisplatin treatment in *Pten/WT* and *Pten/KO* Renca cells in hypoxia; values are shown as the mean ± SEM; Student’s *t*-test (* p-value = 0.026, t_6_ = 3.014)

To identify molecular changes in *Pten/KO* cells, the levels of proteins involved in PTEN-related signaling pathways were assessed. In both types of cancer, pAKT accumulated in *Pten/KO* cells (Fig. 3A, B). The inverse effect of *Pten* mutation in melanoma and RCC was demonstrated in p53 and AKT expression. *Pten/KO* Renca cells had higher levels of p53 than WT cells, while in B16 F10 cells, p53 tended to be downregulated after *Pten* knockout (Fig. 3A, C). AKT expression was reduced in *Pten/KO* cells in the kidney cancer model but not in the melanoma model (Fig. 3A, D). Such modifications of p53, AKT, and pAKT expression as a result of *Pten* knockout observed in both types of cancer were also confirmed in vivo in the tumors (Supplementary Figure S2A, D, E).

In addition to intracellular changes, secretory potential, which modulates TME, may also influence distinct drug sensitivity. In the tested models, *Pten* knockout did not cause changes in the secretion of the main proangiogenic factor VEGF-A in vitro (Fig. 3E)—which may correspond to the lack of changes in susceptibility to sunitinib (Supplementary Figure S3A, B). Also, in vivo, the expressions of *Vegfa* and *Vegfr2* (vascular endothelial growth factor receptor 2) were similar in *Pten/WT* and *Pten/KO* tumors in both melanoma and renal cell carcinoma (Supplementary Fig. 4A, B). PAI-1 was also assessed due to its TME-modulating functions, which promote tumor progression [28]. PAI-1 secretion, high in B16 F10 *Pten/WT* cells, was downregulated by *Pten/KO* (Fig. 3F), which corresponds to a lower cisplatin IC50 dose in *Pten* mutations (Fig. 2B). Decreased *Serpine1* (gene encoding PAI-1) expression was also observed in B16 F10 *Pten/KO* tumors compared to the wild-type control (Supplementary Fig. 4C). This was not observed in the Renca model due to low, close to the limit of detection, PAI-1 secretion. However, the induction of PAI-1 secretion by low oxygen tension (Fig. 3G) showed that *Pten/KO* cells secreted more PAI-1 than *Pten/WT* cells, which corresponds to the changes in IC50 doses for cisplatin—higher in Renca *Pten/KO* cells in hypoxic conditions (Fig. 3G–I).

### Pten knockout induces epithelial-to-mesenchymal transition in Renca cells

**Fig. 4:**
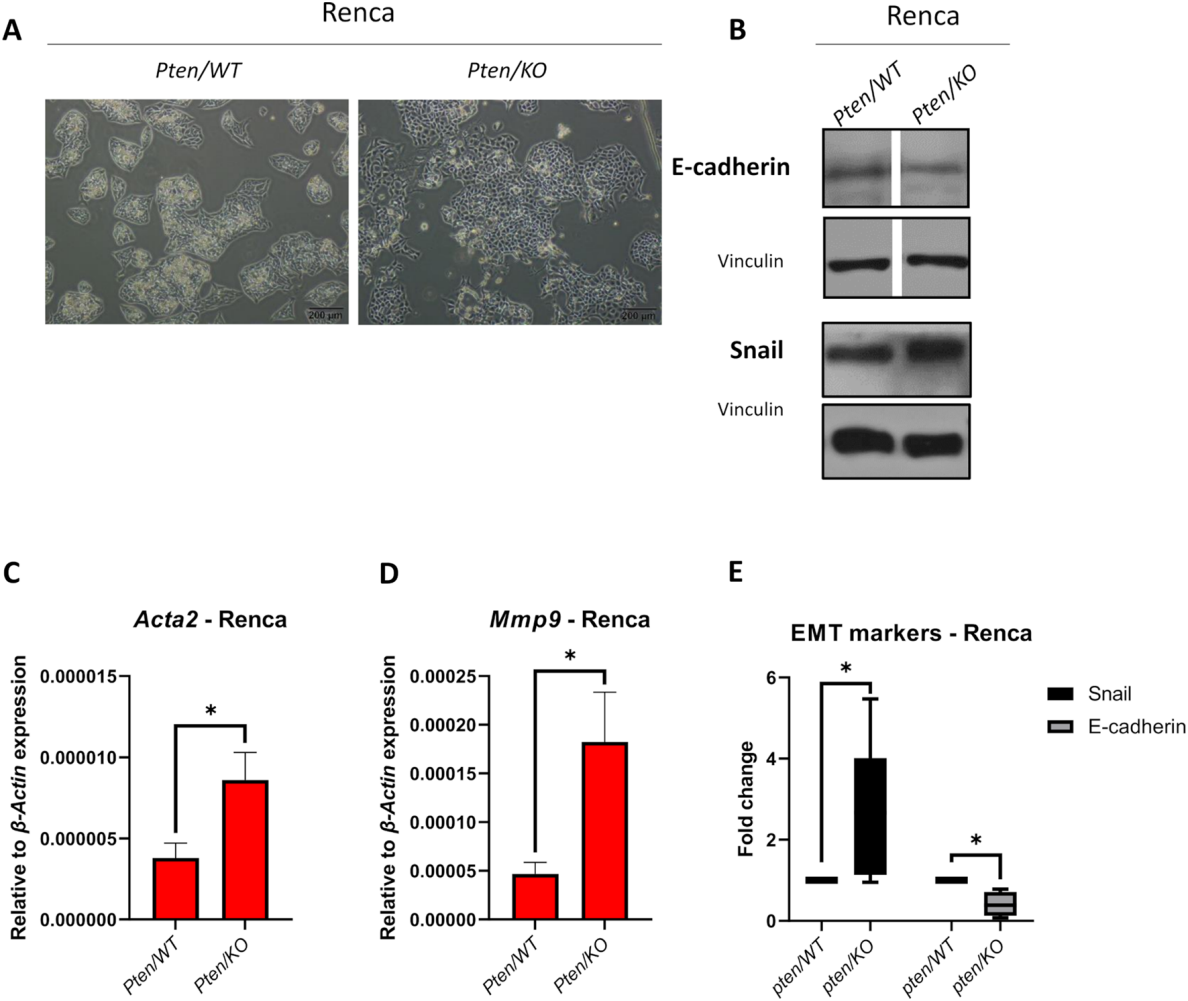
Effect of *Pten* knockout on EMT markers in kidney cancer model. **A** Representative photos showing Renca *Pten/WT* and Pten/KO morphology; scale bar: 200 µm. **B** EMT (epithelial to mesenchymal transition) markers: E-cadherin and Snail detection by western blots with Vinculin as loading control in Renca *Pten/WT* and *Pten/KO* cells. The gap between *Pten/WT* and *Pten/KO* shows that samples on the gel were in a different order and were rearranged for the figure. **C** Expression of *Acta2* (encoding α-SMA) relative to *β-Actin* in Renca *Pten/WT* and *Pten/KO* cells; values are shown as the mean ± SEM; Student’s *t*-test (p-value = 0.0483, t_6_ = 2.472). **D** Expression of *Mmp9* relative to *β-Actin* in Renca *Pten/WT* and *Pten/KO* cells; values are shown as the mean ± SEM; Student’s *t*-test (p-value = 0.0273, t_10_ = 2.583). **E** Box plot represents fold change of Snail and E-cadherin levels relative to Vinculin in *Pten/KO* cell compared to *Pten/WT* cells normalized to 1; middle line in box represents the median; Mann–Whitney U test (Snail: U = 6, *n*_1_ = *n*_2_ = 6, * p-value = 0.0476, two-tailed; E-cadherin: U = 0, *n*_1_ = *n*_2_ = 4, * p-value = 0.0286, two-tailed)

In the kidney cancer model, *Pten* knockout caused changes in cell growth and morphology—cells became more dispersed without forming tight groups (Fig. 4A). This prompted us to investigate the effect of PTEN downregulation on the induction of epithelial-to-mesenchymal transition (EMT). *Pten/KO* Renca cells were characterized by a lower level of E-cadherin with a simultaneous increase in Snail level (Fig. 4B, E). Compared to *Pten/WT* cells, the expression of *Acta2* (gene encoding α-SMA; smooth muscle alpha-actin) and *Mmp9 (*matrix metalloprotease 9) was upregulated in *Pten/KO* cells (Fig. 4C, D). Changed gene expression was also confirmed in vivo; however, E-cadherin and Snail protein levels did not reach a statistically significant level of change in the tumor mass (Supplementary Figure S5A–D).

## Discussion

PTEN is a tumor suppressor [29, 30] that is among the most often mutated genes in cancers [2]; however, its prognostic value in most cancers remains debatable [31]. Here, we characterized the effect of *Pten* knockout in two different cancer cell models that were shown previously to differ in PTEN-related regulation [32]. PTEN is a master regulator of several cellular processes; it controls proliferation, migration, and apoptosis [6]. However, in our models, despite pAKT accumulation, PTEN-lacking cells were not significantly different functionally from WT cells. We observed that the lack of PTEN does not influence cell proliferation, clonogenicity in vitro, or, importantly, tumor growth in vivo. In many models, such as breast cancer, glioma, and colon cancer, it has been observed that *PTEN*-mutated cells proliferate faster [33–36]; however, in other models, it has been reported that PTEN status does not always alter proliferation [37]. It is worth noting that in some models, PTEN loss causes growth arrest [38], related to senescence; therefore, the functional effect of *PTEN* knockout is strongly dependent on the cell type. In our model, no effect on cell growth was observed in anchorage-independent growth in the clonogenic assay. In mammary carcinoma and prostate cancer, the depletion of PTEN leads to the increased formation of colonies in terms of their size and/or number [39–41]. In our case, there was a tendency (p-value = 0.2) for *Pten/KO* cells to form larger colonies; however, no change was observed in their number. Therefore, our study confirms that heterogenous cell responses to PTEN loss are dependent upon the type of cancer.

Because PTEN downregulation did not alter cancer progression in the tested models, we examined other mechanisms that could be affected. PTEN is a prognostic factor of treatment response in cancer patients, and it has been observed that PTEN status affects tumor sensitivity to drugs [42]. Indeed, we observed that *Pten* mutated cells responded differently to some chemotherapeutics; however, again, the response was not uniform. Out of both tested drugs, only resistance to cisplatin was significantly affected by PTEN status, but, importantly, the effect was inverse in RCC and melanoma cells. In Renca cells, PTEN loss increased cell resistance to the drug, with IC50 values being over twice those of WT cells.

This is in accordance with observations carried out in ovarian cancer, where cells with high PTEN levels were sensitive to cisplatin treatment [43]. However, melanoma cells were more sensitive to the drug after PTEN depletion. To explain the differential effect of PTEN inactivation on the cells, we checked the level of p53, since it was shown that p53 is required for cisplatin toxicity in PTEN-overexpressing cells [43]. The effect of *Pten* knockout on p53 expression was inverse in RCC and melanoma, similar to cisplatin resistance. However, sensitized melanoma cells tended to downregulate p53, while desensitized RCC cells increased this protein. Although both cell lines carry functional p53 [44, 45], the mechanism of PTEN and p53-mediated response to cisplatin was different from that previously reported in ovarian cells. It may be that the different reactions of cells to PTEN loss can be related to their starting sensitivity to cisplatin. The IC50 value of the WT melanoma cells was over 20 µM and 10 times smaller for the RCC cells; therefore, we could treat B16 F10 cells as intrinsically resistant, while Renca was sensitive to cisplatin.

As p53 expression could not explain cisplatin sensitivity, other mechanisms of drug resistance were examined for their possible modulation by *Pten* knockout. The complex tumor microenvironment and, thus, cancer development can be diversely shaped by factors secreted by cells of different origins that compose the tumor tissue. We evaluated the levels of VEGF, as the major angiogenic factor, and PAI-1, an extracellular matrix (ECM)-regulating protein affecting, among others, cell survival, migration, and invasion [46]. The production of VEGF-A was not altered by PTEN dysregulation, either in highly proangiogenic RCC cells or VEGF-low-secreting melanoma cells. It has been established in other models that PTEN regulates VEGF expression through the control of the AKT/HIF-1α pathway [47]. Lack of regulation of the VEGF pathway could partly explain no observed effect of PTEN loss on resistance to antiangiogenic therapy in our model. Other studies have shown that *Pten* knockout promotes RCC cell resistance to sunitinib and sorafenib in vitro [25], while in our study, in murine RCC, there was no effect on TKI sensitivity.

However, in our study, PTEN dysregulation caused a very strong decrease in PAI-1 secretion in melanoma cells. This effect could not be observed in RCC cells, which are poor PAI-1 secretors. Nonetheless, when exposed to hypoxia, the production of PAI-1 was induced in RCC cells and was potentiated by *Pten* knockout. Therefore, an inverse reaction of melanoma and RCC cells was evidenced again, similar to cisplatin resistance. These phenomena could be related; indeed, it has been observed that both overexpression and addition of recombinant PAI-1 protect cancer cells from cisplatin-induced apoptosis [48, 49]. A product of the *Serpine1* gene, PAI-1 is a member of the serine protease inhibitor family and a key modulator of the plasminogen/plasminase system [50]. It has also been reported to play a role in cancer; it induces tumor migration, invasion, and angiogenesis, and thereby promotes the progression and metastasis of tumors. However, the specific molecular mechanisms underlying the role of PAI-1 in cancer remain insufficiently documented. In our study, melanoma cells, characterized by high IC50 cisplatin values, displayed high basic PAI-1 secretion. Upon PTEN loss, Serpine1 production was halted, which could mediate reduced cisplatin resistance, as observed for paclitaxel [51].

Renca cells, which are sensitive to cisplatin, have low background PAI-1 expression, and PTEN dysregulation did not alter it. However, it has been shown that cisplatin and carboplatin treatment can induce the secretion of this protein, both by cancer and stromal cells [49, 52]. Here, we observed that hypoxia increased the production of PAI-1, which was accompanied by increased cisplatin IC50 values. The effects of hypoxia and *Pten* knockout were additive both in the case of cisplatin resistance and in PAI-1 production. Therefore, our results suggest that the differential effects of *Pten* knockout on drug resistance might be related to distinct *Serpine1* regulation, although we could not explain the background of this phenomenon. It has previously been reported that Serpine1 modulates the AKT/PI3K/PTEN pathway and that PAI-1 loss causes the activation of AKT and the inactivation of PTEN [31]. Consequently, our results suggest that the status of PTEN and, thus, AKT may reciprocally affect PAI-1 regulation.

EMT is a fundamental mechanism of cancer resistance that can be induced by PTEN modification and the regulation of drug response. In breast cancer, it has been reported that *Pten* knockout induces more epithelial phenotypes in vitro, although the cells migrated more actively than WT cells [39]. In the case of our model, *Pten* knockout RCC cells acquired more mesenchymal phenotypes—there was a reduced expression of E-cadherin with a concomitant increase in EMT markers (Snail, *Mmp9,* and *Acta2*), which was also partly maintained in tumors in vivo. Our data are in concordance with observations showing that PTEN downregulation leads to EMT [53]. Additionally, it was observed that PTEN-loss-mediated EMT causes upregulation of cancer stem cell (CSC) populations within tumor cells [54], which could mediate the reduced sensitivity to cisplatin, as CSCs are largely responsible for drug resistance in cancers [55]. Therefore, it may be that *Pten* knockout induced cisplatin resistance in Renca cells by EMT induction and the protective secretion of PAI-1.

## Conclusions

Our data show the diversity of cell responses to PTEN loss. Although tumor growth was unaffected in both cell models, drug sensitivity was modulated differently by *Pten* mutations in RCC and melanoma cells. We showed that *Pten* knockout can alter the cell microenvironment by regulating secreted factors, including PAI-1, which could explain the differential cell reactions to drug treatment. Additionally, PTEN loss causes EMT features in RCC cells that could contribute to cisplatin resistance.

### Supplementary Information

Below is the link to the electronic supplementary material.

Supplementary file1 (PDF 1303 KB)

Supplementary file2 (PDF 1718 KB)

## Data Availability

All data presented in this manuscript (raw and analyzed results) are available upon request from the corresponding author.

## Abbreviations

CSC: Cancer stem cell
ECM: Extracellular Matrix
EMT: Epithelial to mesenchymal transition
IC50: Half-maximal inhibitory concentration
KO: Knockout
NSCLC: Non-small cell lung cancer
Mmp9: Matrix metalloprotease 9
PAI-1: Plasminogen activator inhibitor 1
PD-1: Programmed Death-1
PTEN: Phosphatase and tensin homolog deleted from chromosome 10
RCC: Renal cell carcinoma
TME: Tumor microenvironment
TKI: Tyrosine kinase inhibitor
VEGF-A: Vascular endothelial growth factor A
VEGFR2: Vascular endothelial growth factor receptor 2
WT: Wild type
α-SMA: Smooth muscle alpha-actin
