# Supplementary material for: Pten knockout affects drug resistance differently in melanoma and kidney cancer
Source: Pharmacol Rep. 2023 Sep 6;75(5):1187–99. doi: 10.1007/s43440-023-00523-y (PMC10539195; doi:10.1007/s43440-023-00523-y)
Supplement: Supplementary file 1 — Supplementary file1 (PDF 1303 KB) [file 43440_2023_523_MOESM1_ESM.pdf]

Pten knockout affects drug resistance differently in melanoma and kidney cancer

Klaudia Brodaczewska [1, *] , Aleksandra Majewska [1, 2, *] , Aleksandra Filipiak-Duliban [1, 2], Claudine Kieda [1, 3]

[1] Military Institute of Medicine – National Research Institute, Laboratory of Molecular Oncology and Innovative Therapies, Szaserów 128, 01-141 Warsaw, Poland

[2] Postgraduate School of Molecular Medicine (Medical University of Warsaw), Żwirki i Wigury 61, 02-091 Warsaw, Poland

[3] Center for Molecular Biophysics UPR 4301 CNRS, 45071 Orleans, France

* These authors contributed equally to this work

Corresponding author: Klaudia Brodaczewska; kbrodaczewska@wim.mil.pl; Szaserów 128, 01-141 Warsaw, Poland

SUPPLEMENTARY MATERIALS

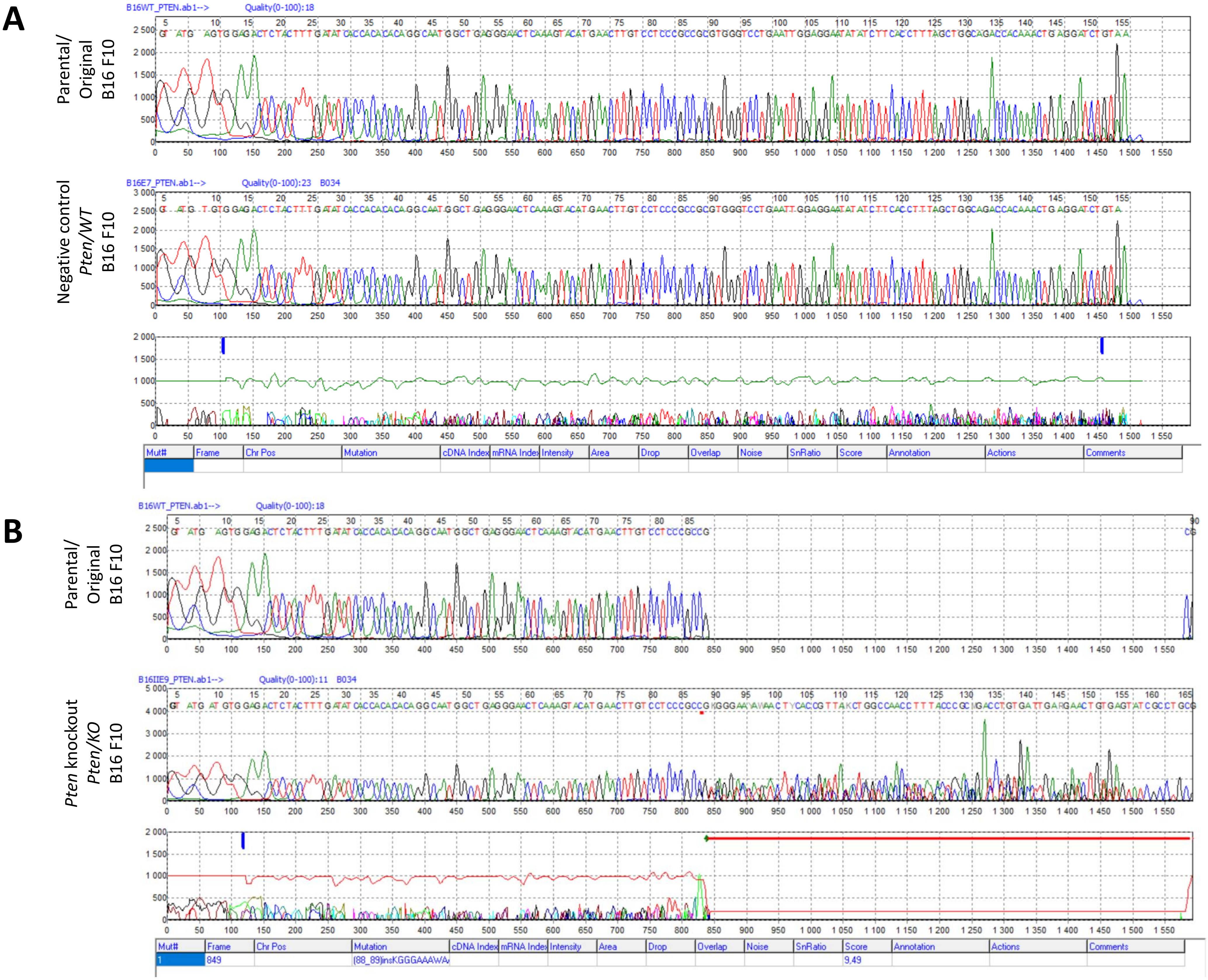

C

No.	Sample File	Reference File	Dir	Gene	Exon	RF	Start	End	Size	Qualit	Mut#	Mutation1	Mutation2
1	B16E7_PTEN.ab1	B16WT_PTEN.ab1	1				(8)	(149)	142	23	0		
2	B16IIE9_PTEN.ab1	B16WT_PTEN.ab1	1				(12)	(153)	142	11	2	(87)C>CG$16	(88_89)insKGGG
2									284	17	1,00	50,0%	50,0%

Figure S1. **Sequencing results.**
(A) Comparison of parental/original B16 F10 cell line to *Pten/WT* B16 F10 cells – negative control. (B) Comparison of parental/original B16 F10 cell line to *Pten/KO* B16 F10 cells – model of *Pten* knockout. (C) Summary of mutation analysis.

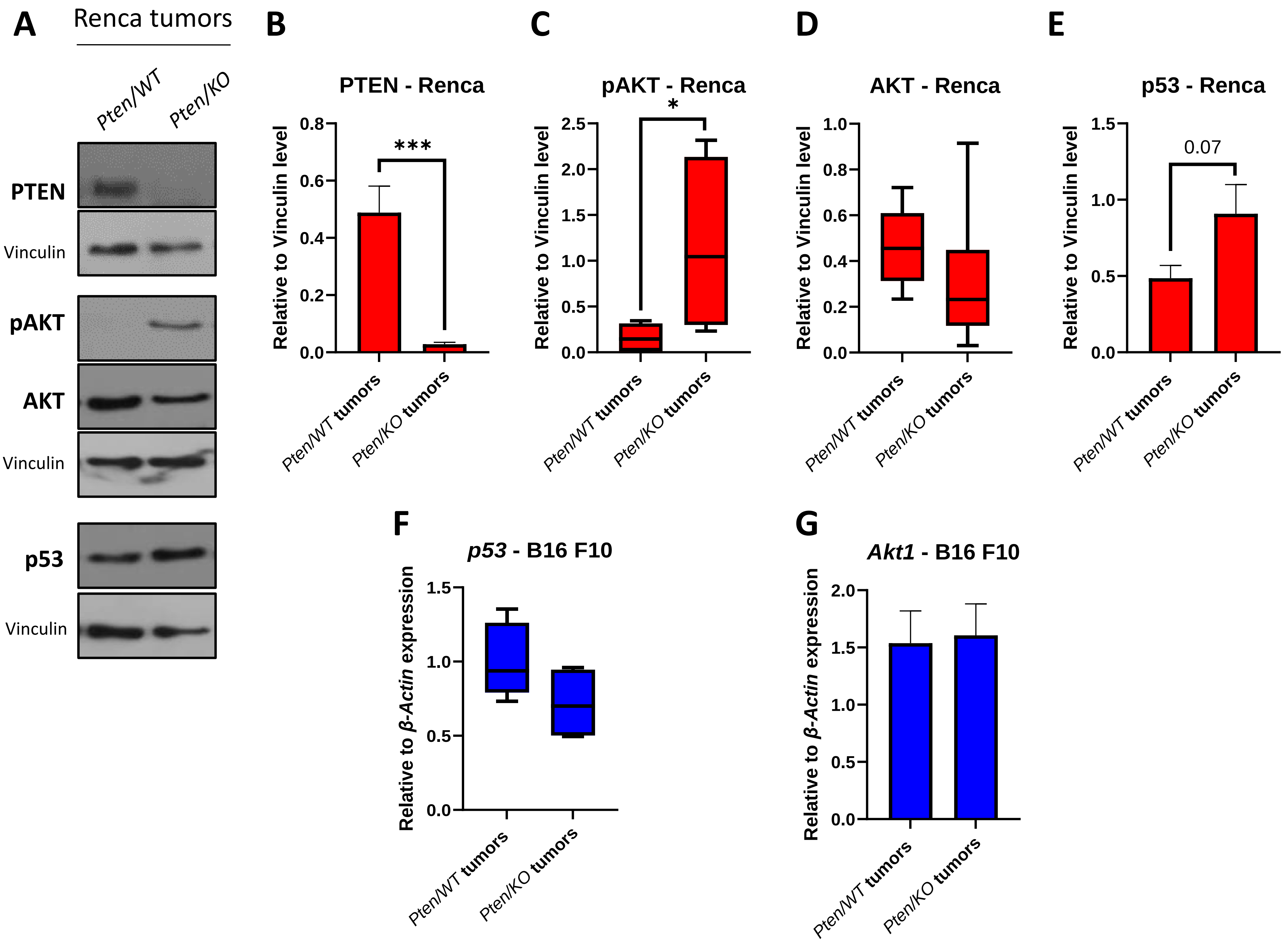

Figure S2. **Molecular changes in B16 F10 and Renca tumors with different PTEN status.**
(A) PTEN, p-AKT, AKT and p53 detection by western blots with Vinculin as loading control in Renca tumors with different PTEN statuses. (B) PTEN level relative to Vinculin in Renca *Pten/WT* and *Pten/KO* tumors mass; values are shown as the mean ± SEM; Student's *t*-test (*** p-value = 0.0006, t_{10} = 4.928). (C) Box-plot of relative to Vinculin pAKT level in Renca *Pten/WT* and *Pten/KO* tumors mass; middle line in box represents the median; Mann–Whitney U test (U = 4, n_1 = n_2 = 6, * p-value = 0.0260, two-tailed). (D) Box-plot of relative to Vinculin AKT level in Renca *Pten/WT* and *Pten/KO* tumors mass; middle line in box represents the median; Mann-Whitney U test (U = 8, n_1 = n_2 = 6, p-value = 0.1320, two-tailed). (E) p53 level relative to Vinculin in Renca *Pten/WT* and *Pten/KO* tumors mass; values are shown as the mean ± SEM; Student's *t*-test (p-value = 0.0707, t_{10} = 2.023). (F) Box-plot of relative to *β-Actin* expression of *p53* in B16 F10 *Pten/WT* and *Pten/KO* tumors mass; middle line in box represents the median; Mann–Whitney U test (U = 9, n_1 = n_2 = 6, p-value = 0.1797, two-tailed). (G) Relative to *β-Actin* expression of *Akt1* in B16 F10 *Pten/WT* and *Pten/KO* tumors mass; values are shown as the mean ± SEM; Student's *t*-test (p-value = 0.8641, t_{10} = 0.1756).

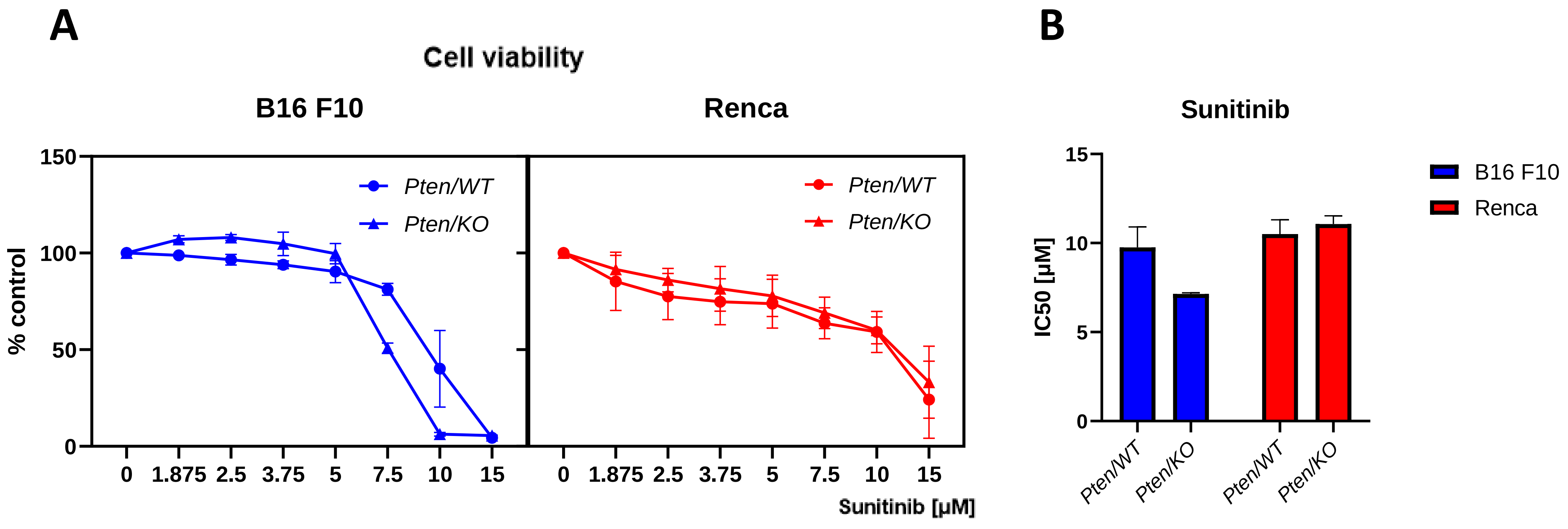

Figure S3. **Effect of *Pten* knockout on sunitinib sensitivity in B16 F10 and Renca cells.**
(A) Viability of B16 F10 and Renca cells with different PTEN statuses after various doses of sunitinib (Sigmaaldrich, Darmstadt, Germany) treatment, measured by Alamar Blue, shown as a percentage of untreated control for each PTEN variant normalized to 100 %; B16 F10 n = 4, Renca n = 3. (B) IC50 dose (half-maximal inhibitory concentration) of sunitinib treatment for different PTEN variant cells; values are shown as the mean ± SEM; Student's *t*-test (B16 F10: p-value = 0.0627, t_6 = 2.281; Renca p-value = 0.5745, t_4 = 0.6105).

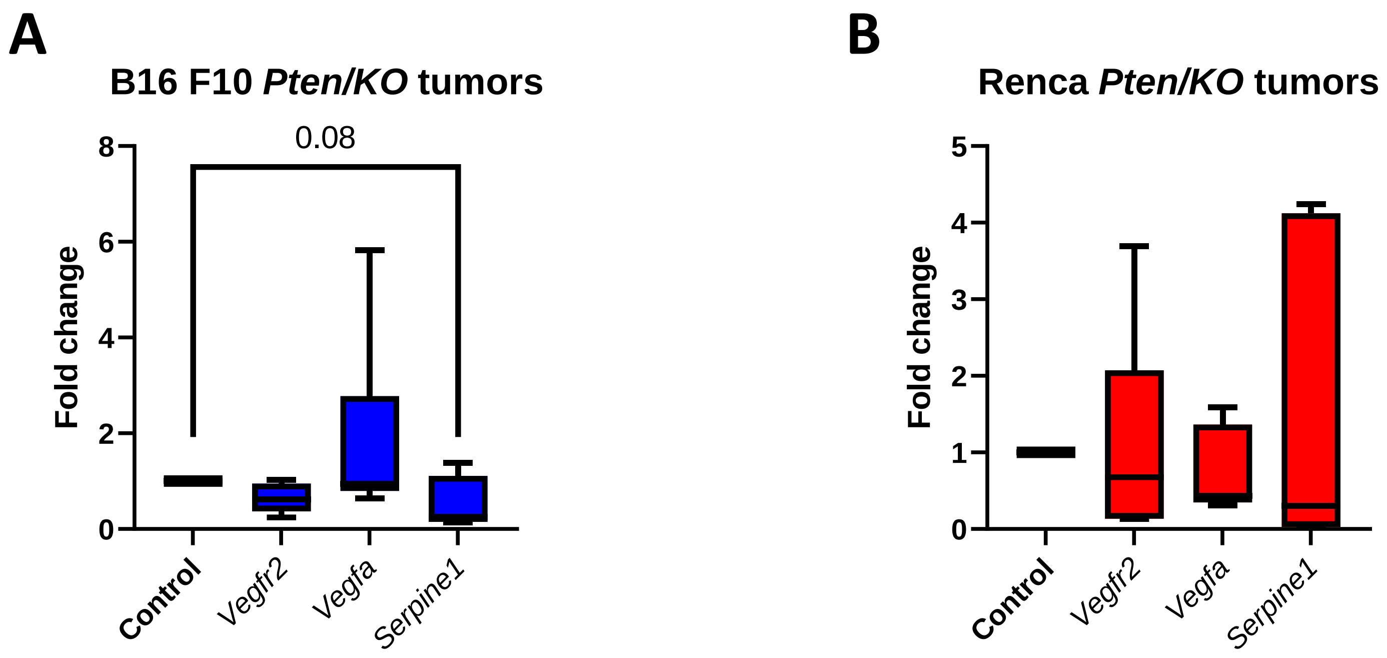

Figure S4. **Molecular changes in B16 F10 and Renca tumors with different PTEN status**.
(A) Box plot represents fold change of relative to *β-Actin Vegfr2*, *Vegfa*, and *Serpine1* expression in B16 F10 *Pten/KO* tumors, compared to Control - *Pten/WT* tumors normalized to 1*;* middle line in box represents the median; Mann–Whitney U test (*Vegfr2*: U = 5, *n* = 5, * p-value = 0.1270, two-tailed; *Vegfa:* U = 12, *n* = 6, * p-value = 0.3636; *Serpine1:* U = 6, *n* = 5, * p-value = 0.0801). (B) Box plot represents fold change of relative to *β-Actin Vegfr2*, *Vegfa*, and *Serpine1* expression in Renca *Pten/KO* tumors, compared to Renca *Pten/WT* tumors normalized to 1*;* middle line in box represents the median; Mann–Whitney U test (not significant).

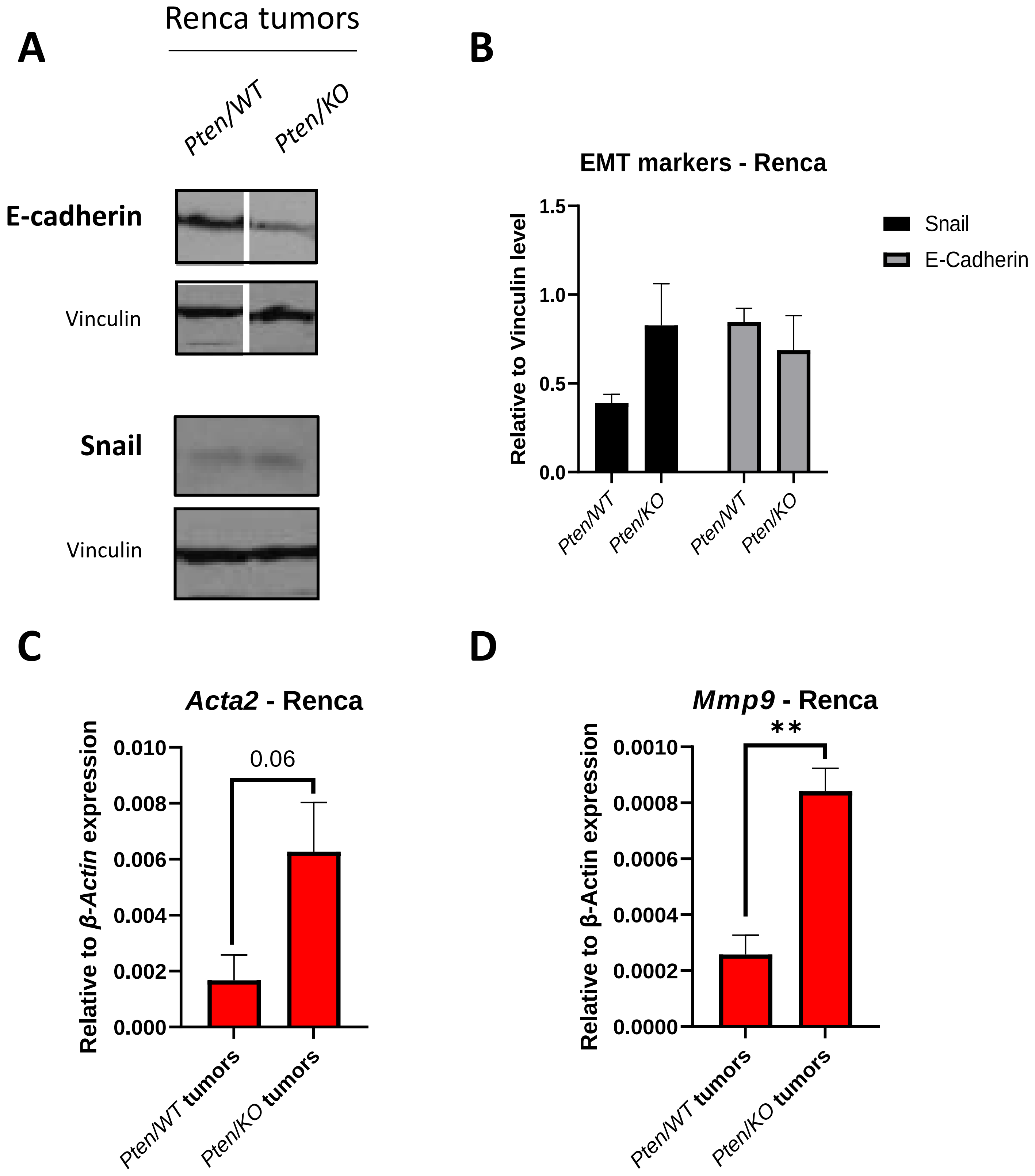

Figure S5. Expression of EMT markers in Renca tumors with different PTEN status.
(A) Epithelial to mesenchymal transition (EMT) markers: E-cadherin and Snail detection by western blots with Vinculin as loading control in Renca tumors with different PTEN status. The gap between *Pten/WT* and *Pten/KO* shows that samples on the gel were in a different order and were rearranged for the figure. (B) E-cadherin and Snail levels relative to Vinculin in Renca *Pten/WT* and *Pten/KO* tumor masses; values are shown as the mean ± SEM; Student's *t*-test (not significant). (C) Relative to *β-Actin* expression of *Acta2* (encoding α-SMA) in Renca *Pten/WT* and *Pten/KO* tumor masses; values are shown as the mean ± SEM; Student's *t*-test (p-value = 0.0594, t_6 = 2.321). (D) Relative to *β-Actin* expression of *Mmp9* in Renca *Pten/WT* and *Pten/KO* tumor masses; values are shown as the mean ± SEM; Student's *t*-test (** p-value = 0.0016, t_6 = 5.457).
