# Supplementary material for: Pten knockout affects drug resistance differently in melanoma and kidney cancer
Source: Pharmacol Rep. 2023 Sep 6;75(5):1187–99. doi: 10.1007/s43440-023-00523-y (PMC10539195; doi:10.1007/s43440-023-00523-y)

The membranes were cut according to the size of checked proteins.

1A

1B

1C

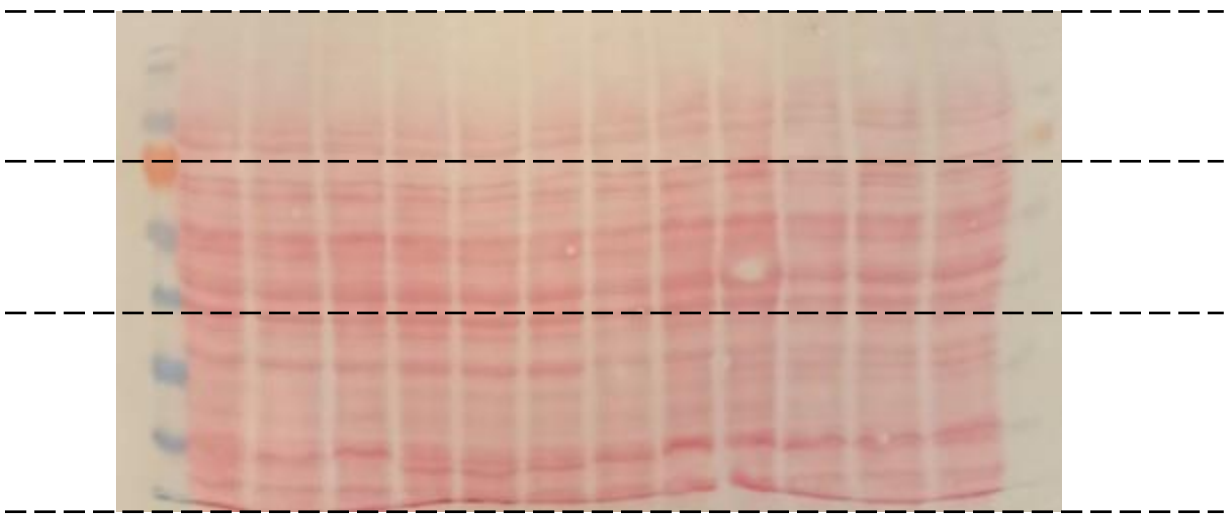

1) Each part od membrane were incubated in different primary antibodies.
 eg. 1A incubation in anti-PROTEIN-X antibody (rabbit)
 1B incubation in **anti-PTEN** antibody (**mouse**)
 1C incubation in anti-PROTEIN-Y antibody (mouse)

After incubation each membrane were incubated with secondary antibody (anti-mouse or anti-rabbit depending on origin of primary antibody). Visualization was performer using x-ray films (depending on exposure time: all parts of membrane were visualized on one x-ray film or each part of membrane was visualized on different x-ray film

2) Next, each part of membrane was incubated in primary antibody (different origin)
eg.1A incubation in **anti-Vinculin** antibody (mouse)
 1B incubation in **anti-pAKT** antibody (**rabbit**)
 1C incubation in anti-PROTEIN-Z antibody (rabbit)

After incubation each membrane were incubated with secondary antibody (anti-mouse or anti-rabbit depending on origin of primary antibody). Visualization was performer using x-ray films (depending on exposure time: all parts of membrane were visualized on one x-ray film or each part of membrane was visualized on different x-ray film

## pAKT and PTEN → Both proteins have same Vinculin bands

B16 F10 cell line

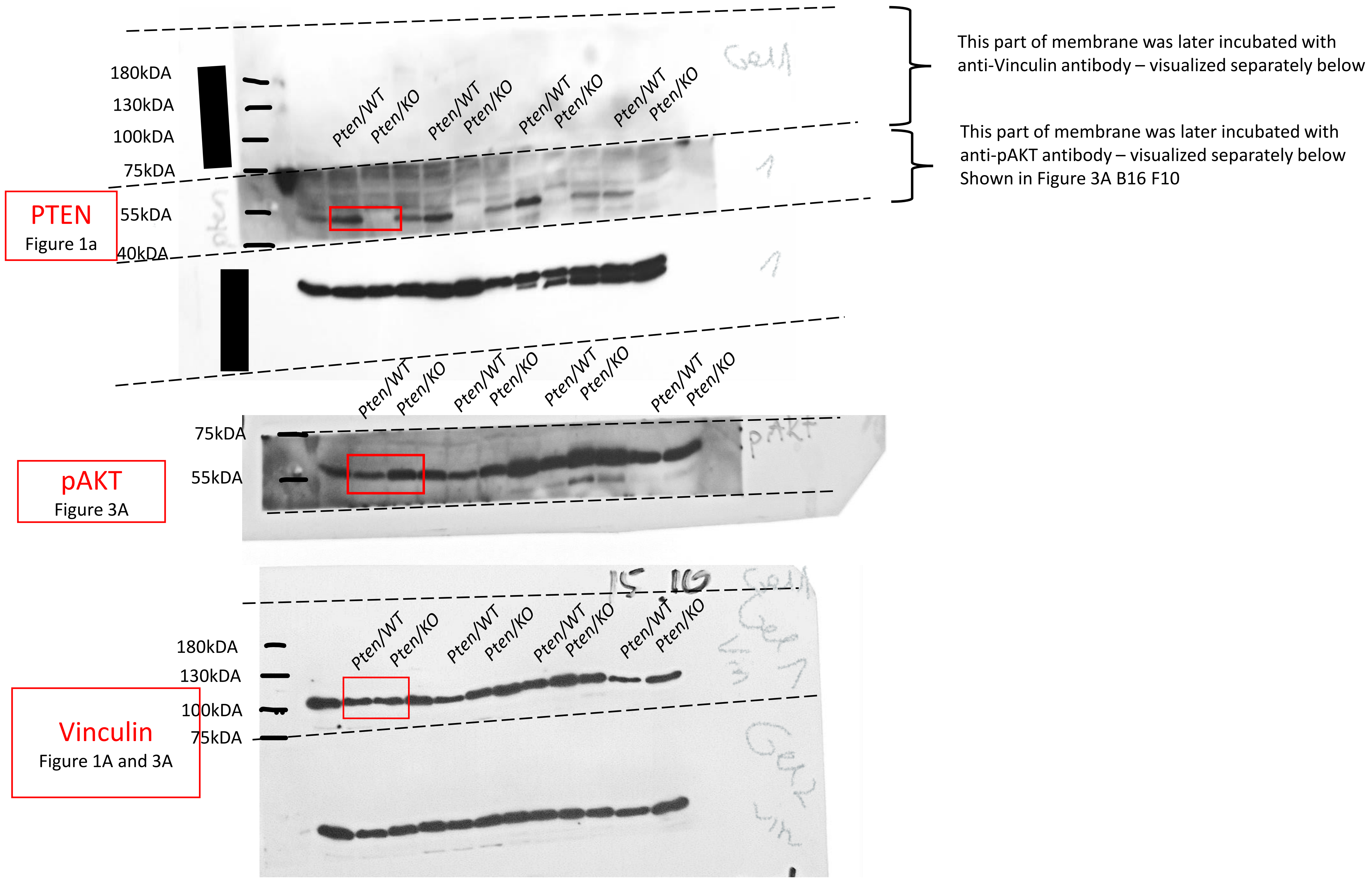

B16 F10 cell line

AKT
Figure3a

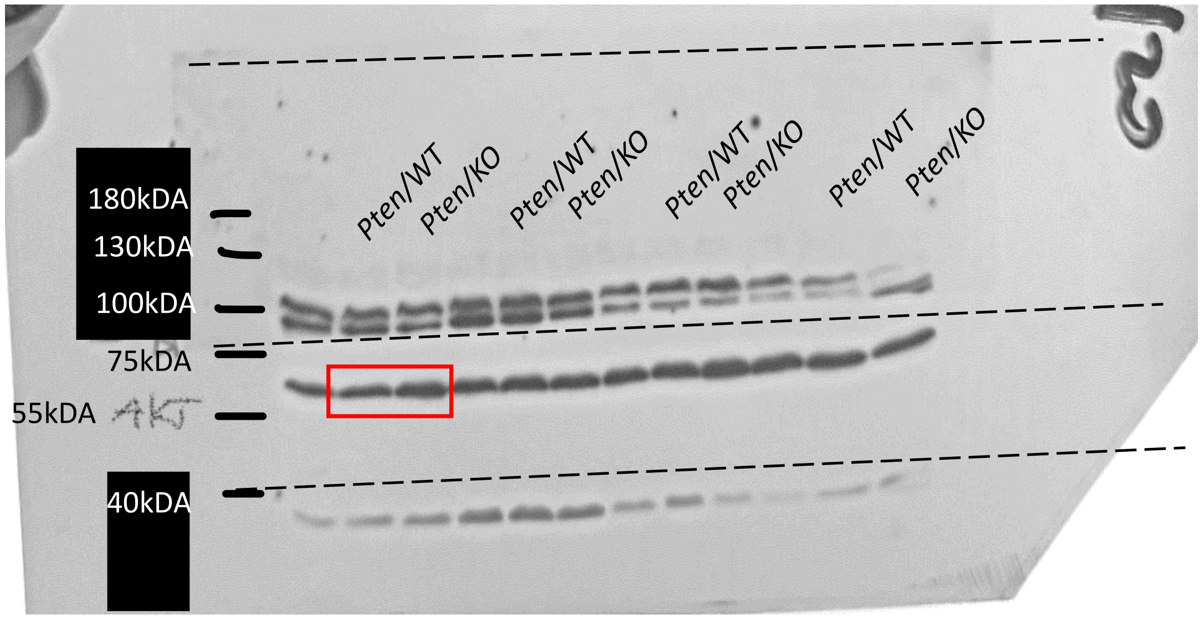


This part of membrane was later incubated with anti-Vinculin antibody – visualized separately below

Vinculin
Figure 3a

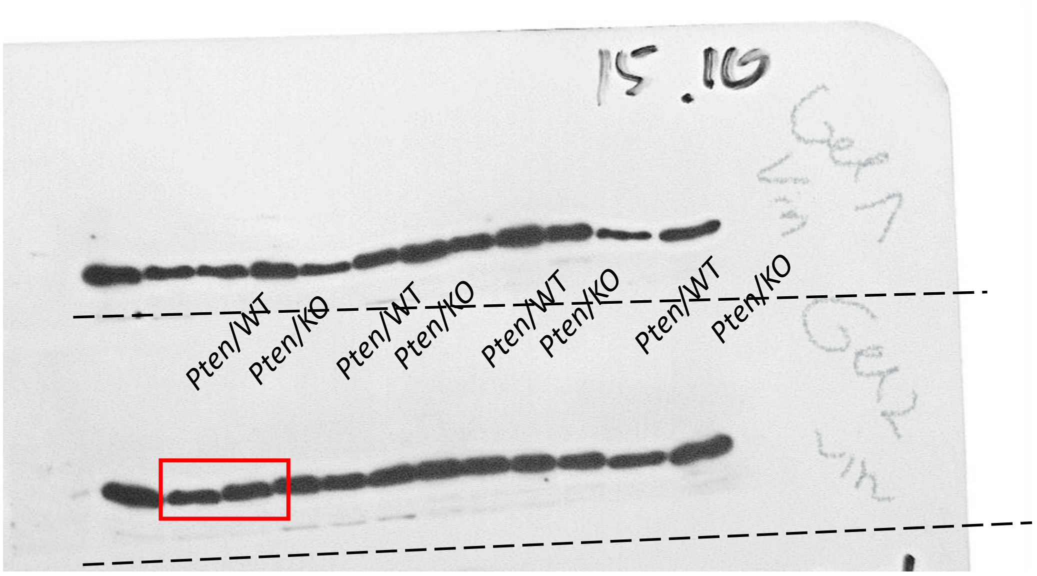

B16 F10 cell line

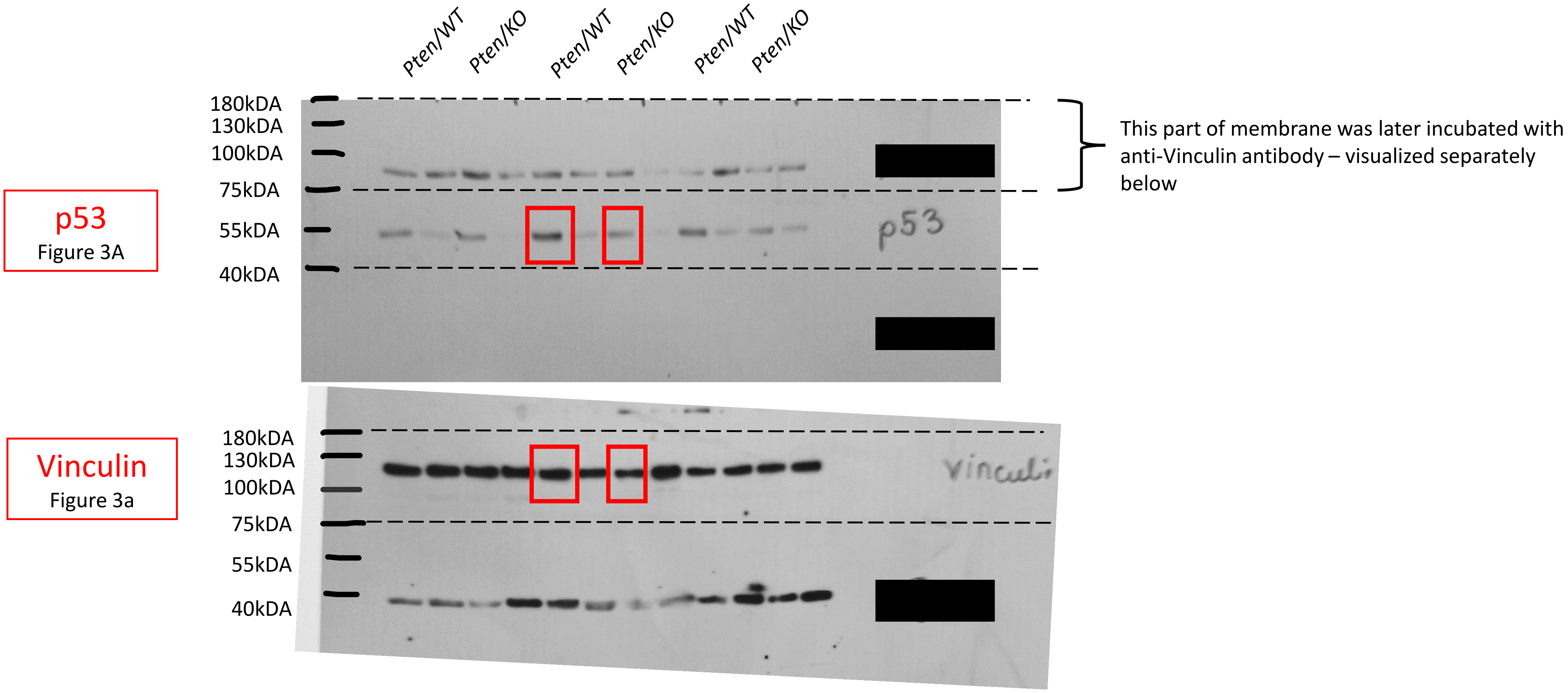

Renca cell line

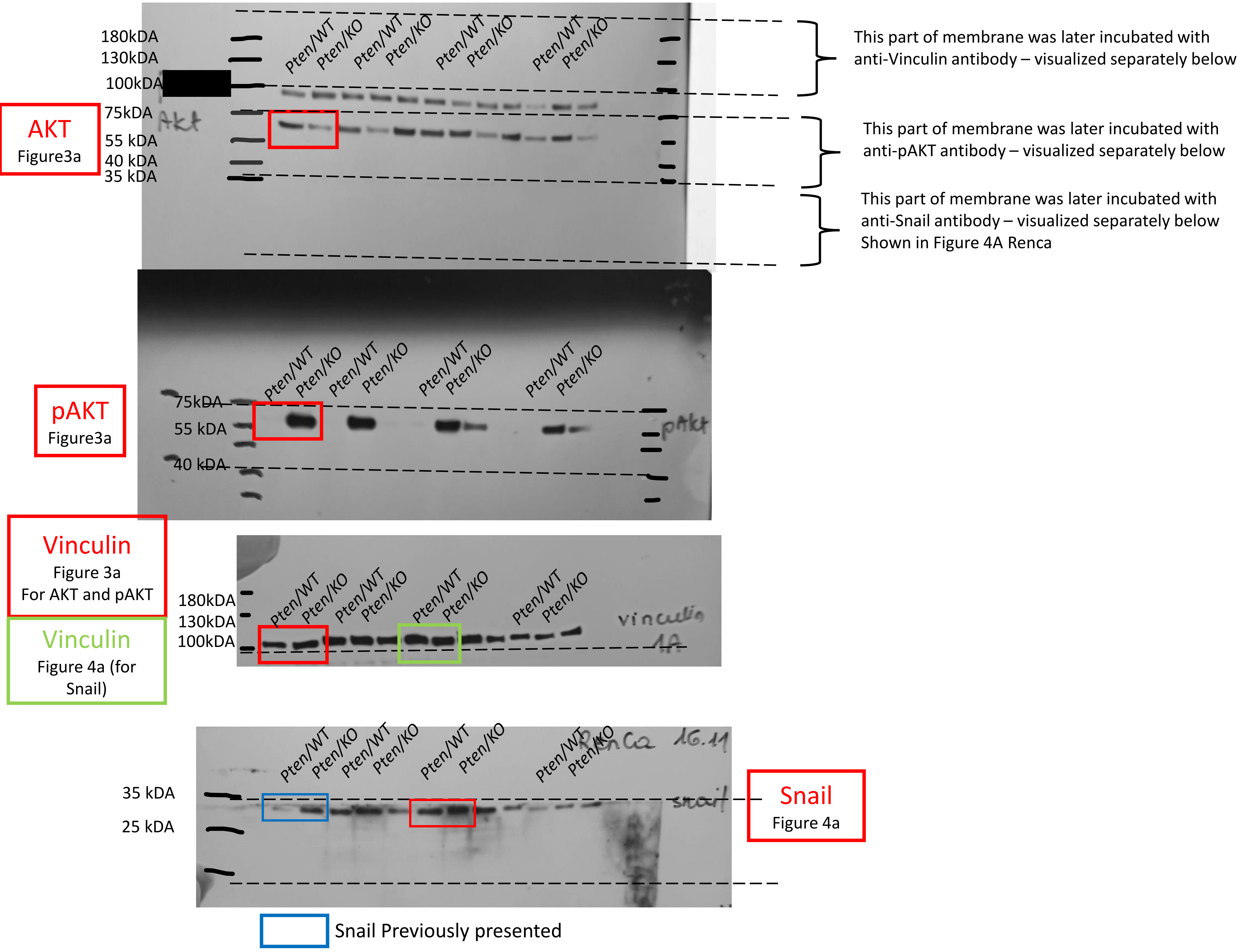

# Renca cell line

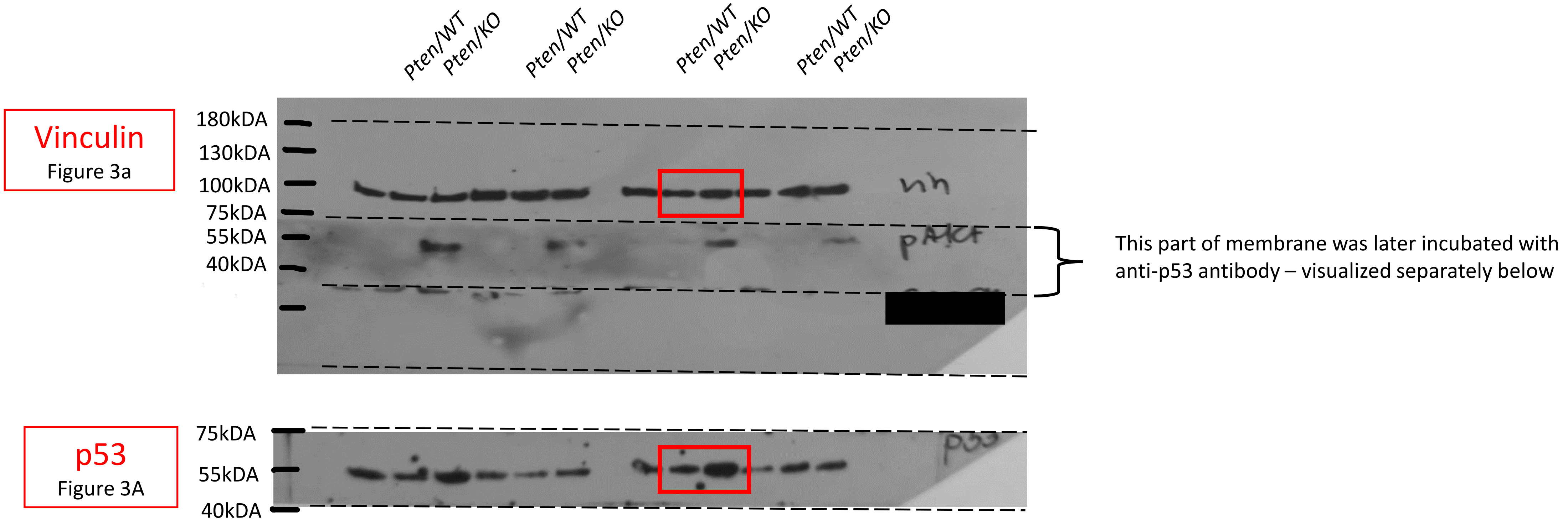

Renca cell line

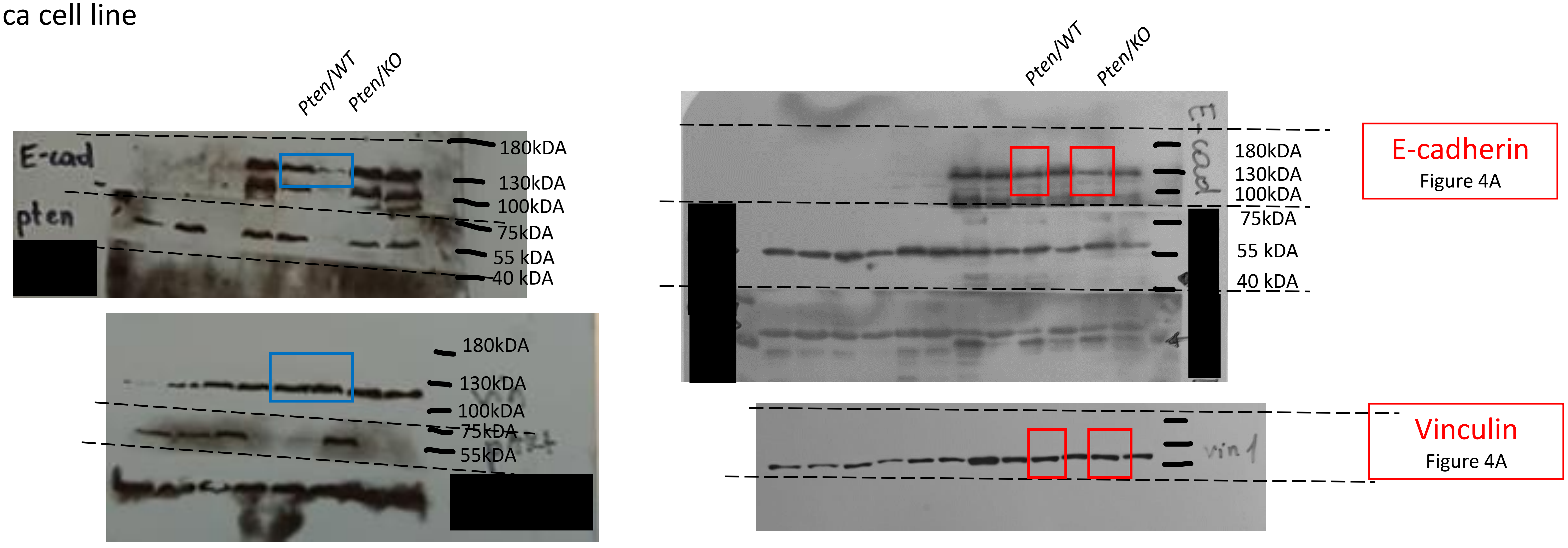


Previously presented

Currently presented

# Renca tumors

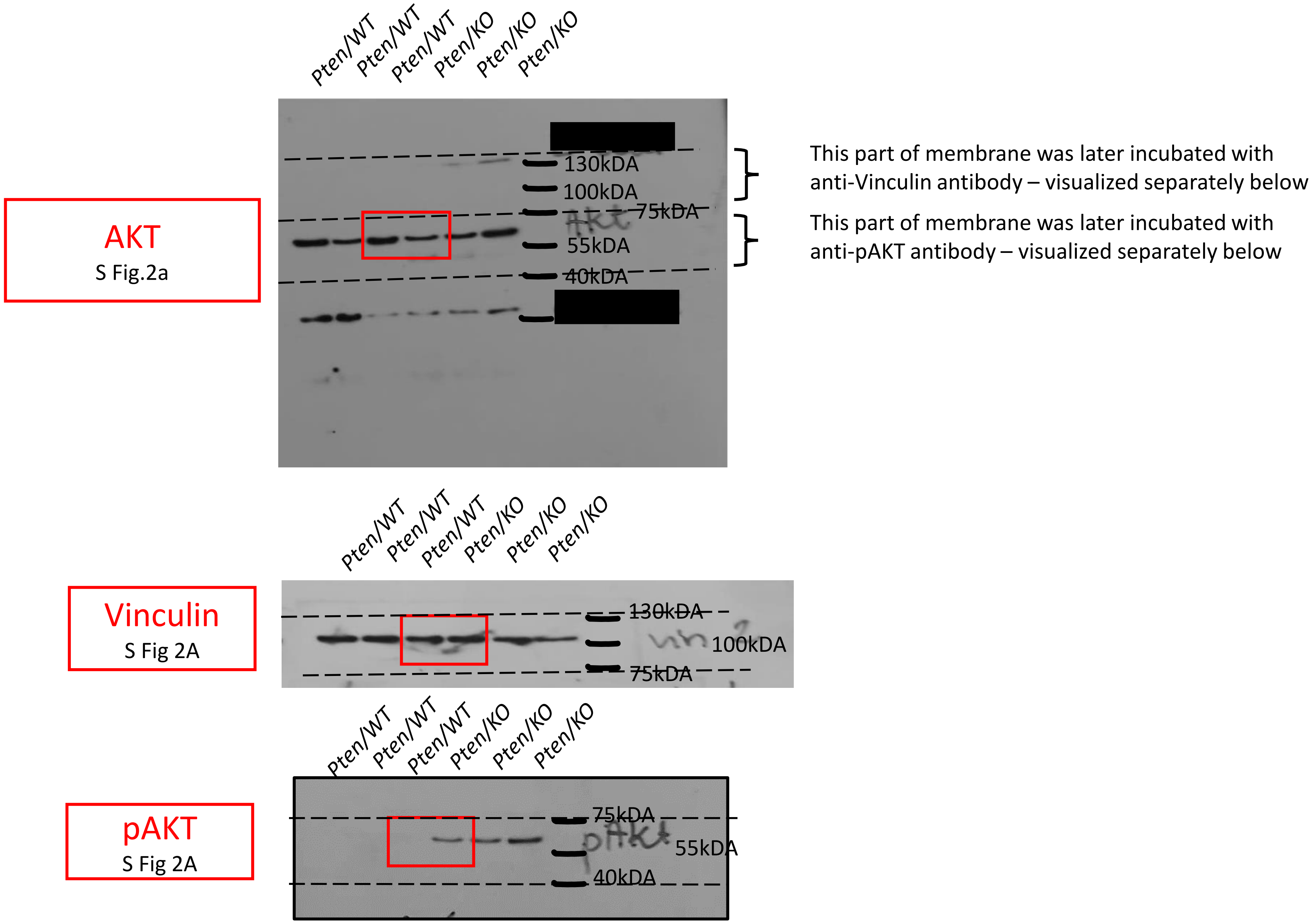

Renca tumors

P53
S Fig 2A

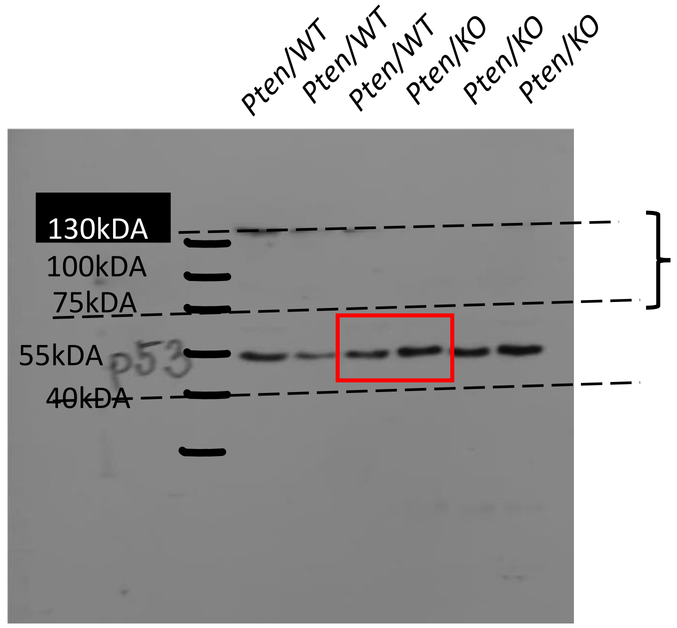


This part of membrane was later incubated with anti-Vinculin antibody – visualized separately below

Vinculin
S Fig 2A

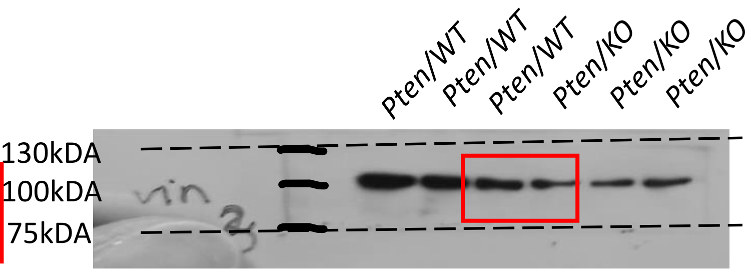


PTEN
S Fig 2A

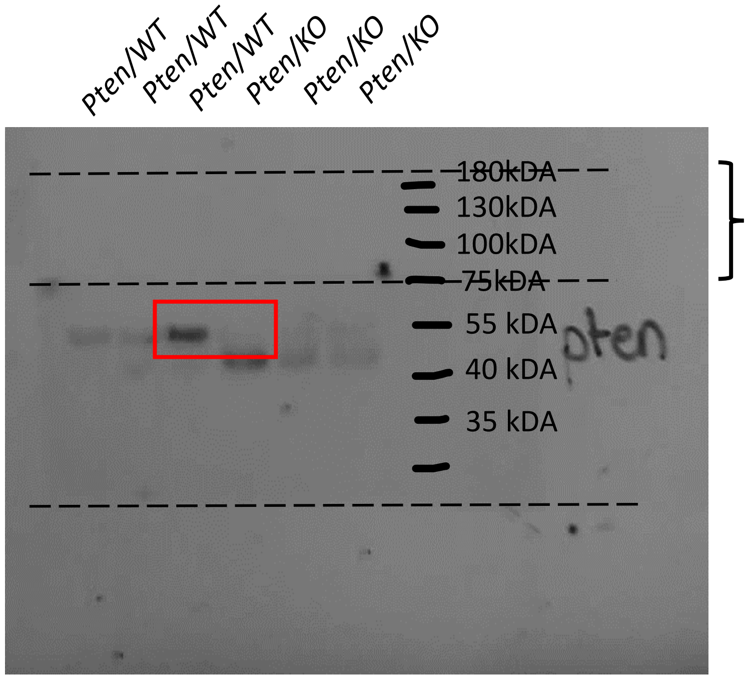


This part of membrane was later incubated with anti-Vinculin antibody – visualized separately below

Vinculin
S Fig 2A

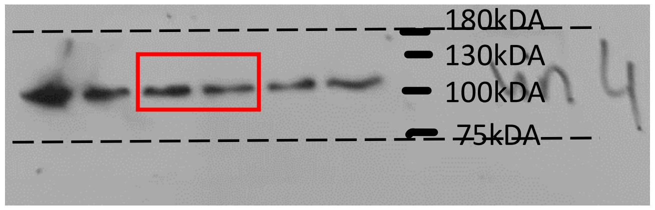

Renca tumors

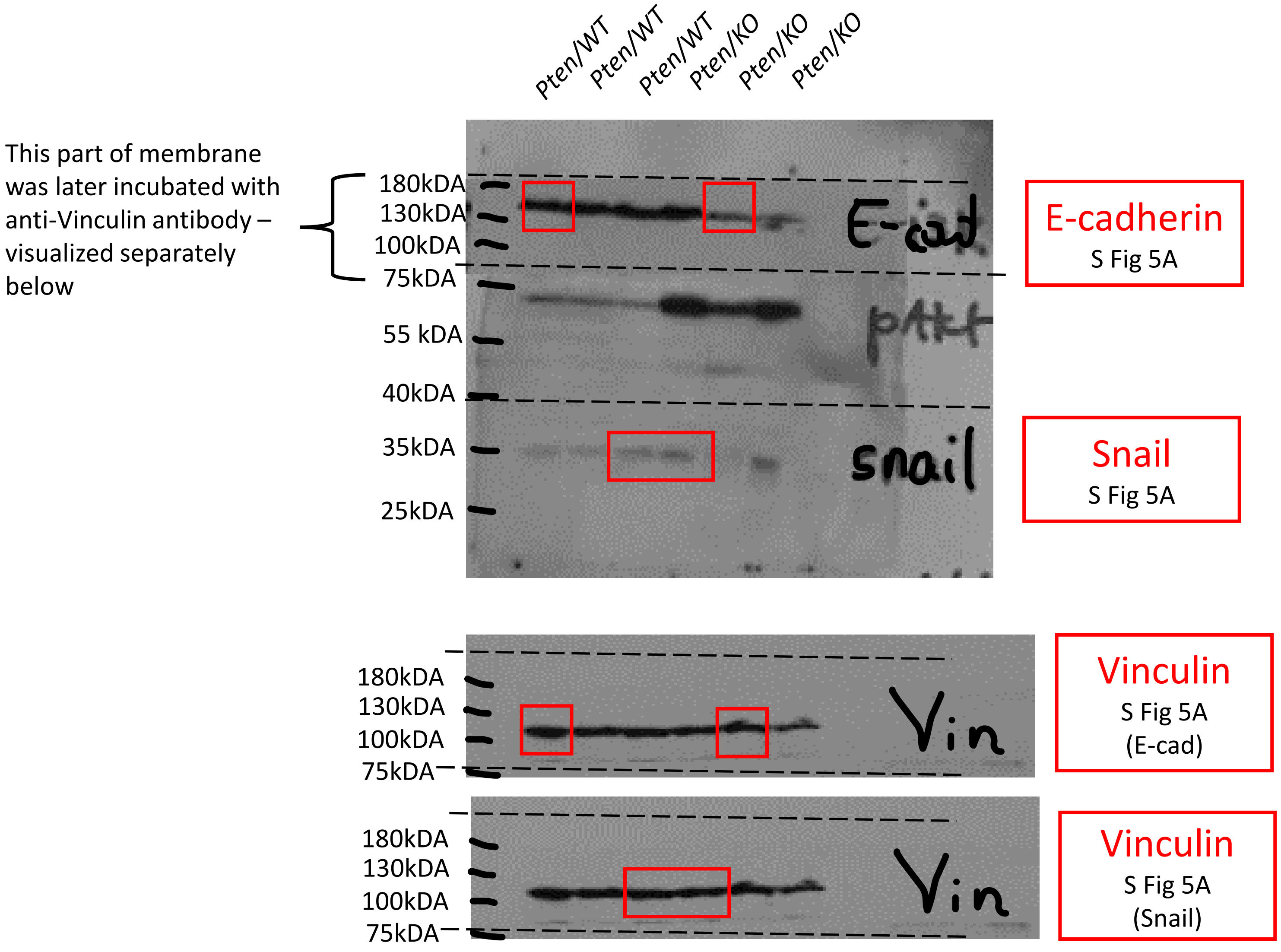

Supplement: Supplementary file 2 — Supplementary file2 (PDF 1718 KB) [file 43440_2023_523_MOESM2_ESM.pdf]
